# Linking differences in microbial network structure with changes in coral larval settlement

**DOI:** 10.1038/s43705-023-00320-x

**Published:** 2023-10-21

**Authors:** Abigail C. Turnlund, Inka Vanwonterghem, Emmanuelle S. Botté, Carly J. Randall, Christine Giuliano, Lisa Kam, Sara Bell, Paul O’Brien, Andrew P. Negri, Nicole S. Webster, Miguel Lurgi

**Affiliations:** 1 The University of Queensland, School of Chemistry and Molecular Biosciences, Australian Centre for Ecogenomics, St Lucia, QLD 4072 Australia; 2 Centre for Marine Science and Innovation, School of Biological, Earth and Environmental Sciences, University of New South Wales, Sydney, NSW Australia; 3 Australian Institute of Marine Science, Townsville, QLD Australia; 4 Department of Climate Change, Energy, the Environment and Water, Australian Antarctic Division, Kingston, ACT Australia; 5 Department of Biosciences, Swansea University, Swansea, SA2 8PP UK

**Keywords:** Water microbiology, Microbial ecology, Symbiosis

## Abstract

Coral cover and recruitment have decreased on reefs worldwide due to climate change-related disturbances. Achieving reliable coral larval settlement under aquaculture conditions is critical for reef restoration programmes; however, this can be challenging due to the lack of reliable and universal larval settlement cues. To investigate the role of microorganisms in coral larval settlement, we undertook a settlement choice experiment with larvae of the coral *Acropora tenuis* and microbial biofilms grown for different periods on the reef and in aquaria. Biofilm community composition across conditioning types and time was profiled using 16S and 18S rRNA gene sequencing. Co-occurrence networks revealed that strong larval settlement correlated with diverse biofilm communities, with specific nodes in the network facilitating connections between modules comprised of low- vs high-settlement communities. Taxa associated with high-settlement communities were identified as *Myxoccales sp*., *Granulosicoccus sp*., *Alcanivoraceae sp*., unassigned JTB23 *sp*. (*Gammaproteobacteria*), and *Pseudovibrio denitrificans*. Meanwhile, taxa closely related to *Reichenbachiella agariperforans, Pleurocapsa sp., Alcanivorax sp., Sneathiella limmimaris*, as well as several diatom and brown algae were associated with low settlement. Our results characterise high-settlement biofilm communities and identify transitionary taxa that may develop settlement-inducing biofilms to improve coral larval settlement in aquaculture.

## Introduction

Ocean warming and associated heatwaves are increasing the frequency of mass coral bleaching events, compromising the health and resilience of coral reefs worldwide [1, 2]. These events are further exacerbated by local stressors including outbreaks of the coral predator Crown-of-Thorns starfish [3] and declining water quality [4]. Coral reef recovery largely depends on the corals’ ability to produce larvae that can successfully settle and survive on reef substrata [5]. Yet in recent decades, coral recruitment success has declined due to decreasing coral cover and loss of suitable substrate [6–8]. Enhancing larval recruitment in reef restoration efforts can help support the long-term survival and persistence of coral reefs [1].

Coral aquaculture, i.e., the cultivation of corals in specialised aquaria, is increasingly being adopted to support large-scale coral reef restoration [1, 3, 6, 9]. Coral cultivation can be achieved via asexual or sexual propagation. The latter provides significant benefits by generating genetic diversity [1] and minimising further harm to adult coral colonies that would have otherwise been damaged through fragmentation [10]. However, a clear bottleneck for coral sexual propagation is the limited understanding of the specific environmental cues underpinning larval settlement and metamorphosis [1]. Moreover, these cues likely vary among coral species [1, 11, 12] and can be of physical, biological and/or chemical nature [13]. To improve coral larval settlement in aquaculture, we need to elucidate these specific cues.

Previous research investigating potential marine invertebrate larval settlement inducers revealed that various species of crustose coralline algae (CCA) and/or their associated microbial biofilms can trigger settlement across a broad range of species [11, 14–17]. In corals, larval choice experiments with CCA-associated biofilms showed that settlement of the coral *Acropora millepora* was negatively correlated with *Flammeovirga sp*. and *Vibrio sp*., but positively correlated with *Neptuniibacter sp*. and a member of the marine Methylotrophic group 3 [14]. Additionally, the organic compound tetrabromopyrrole (TBP), isolated from the bacterial species *Pseudoalteromonas sp. PS5*, has been shown to induce metamorphosis in coral larvae [16–19], however, it is unlikely to contribute to inductive properties of some microbial biofilms [16]. While microorganisms may contribute to the recruitment process of certain coral species, our knowledge of the specific microbial taxa or metabolites that trigger larval recruitment is still limited [1, 15], in part due to experimental constraints. While reef biofilms are comprised of a complex assemblage of prokaryotes [20–24], most research to date has relied on the cultivation of mono-specific biofilms to identify potential inducers of coral larval settlement. Given that culturable microorganisms typically represent <1% of the community [11, 17, 19, 25], this approach considerably reduces the potential to identify specific settlement inducers. Therefore, it is important to combine settlement assays with molecular approaches that allow for a comprehensive understanding of the microbial diversity of marine biofilms to elucidate their involvement in the coral recruitment process. For example, combining high-throughput sequencing data with a network analysis of taxon co-occurrence patterns allows for analysis of community dynamics and identification of inductive taxa, and thus provides insights into the community-assembly mechanisms that drive settlement.

Here, a settlement choice experiment was performed to characterise microbial communities involved in settlement of the corymbose coral *Acropora tenuis*. Coral larvae were exposed to marine biofilms established under aquarium (laboratory) or reef (field) conditions, and larval settlement across the different substrates was measured. Microbial biofilms were subsequently characterised using 16S and 18S rRNA gene sequencing and networks of co-occurrences among microbial taxa were constructed to examine the structure of microbial communities that correlated with various levels of larval settlement. Furthermore, analyses of these networks allowed for the identification of microbial taxa that were integral to community structure through the strength of their connections with other microorganisms and their position within the network.

We hypothesised that marine biofilms would show strong differences in microbial community composition based on establishment conditions, and that specific microbial taxa would be associated with low and high-settlement-inducing biofilms. We identified microbial taxa associated with specific settlement levels that were more commonly found together than with others, creating tight modules within the network, as well as key taxa that serve as links between modules and may be involved in transitioning microbial communities to induce greater coral settlement. Our work identifies potential candidates for coral larval settlement and gives a better understanding of microbial biofilm interactions with coral larvae to improve coral aquaculture for reef restoration.

## Methods

### Experimental design and sampling

**Fig. 1:**
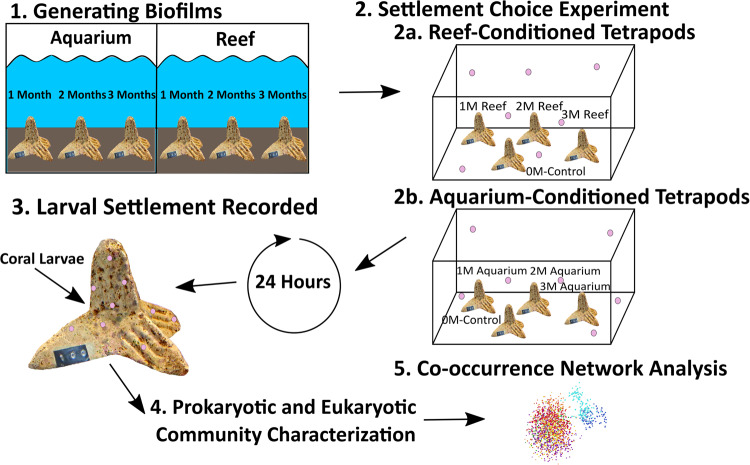
Schematic of the Acropora tenuis larval choice experiment with pre-conditioned biofilms. (1) Blank tetrapods were deployed in both the Australian Institute of Marine Science’s SeaSimulator (subsequently designated ‘aquarium’) and at Backnumbers Reef (subsequently designated ‘reef’) for 1, 2 and 3 months, to establish biofilms. (2) Following coral spawning, tetrapods from each conditioning treatment were placed in respective tanks together (along with a 0-month control) with *A. tenuis* coral larvae for 24 hours. (3) After 24 h, larval settlement was scored for each tetrapod and the biofilms were scraped from the tetrapods for downstream molecular analysis. (4) Microbial communities within the biofilms, larvae and seawater were characterised using 16S and 18S rRNA gene amplicon sequencing. (5) Microbial communities were analysed using co-occurrence network analysis.

Thirty-six concrete tetrahedral blocks (‘tetrapods’) (90 mm × 75 mm), designed by SECORE International, were deployed in aquaria of the National Sea Simulator at the Australian Institute of Marine Science and at Backnumbers Reef, GBR (18°29′25.00″S, 147° 9′18.00″E) for conditioning periods of 1, 2 or 3 months for biofilm development (from September 2018 to November 2018). Reef-conditioned tetrapods were deployed over a 3-month period at ~6 m depth in rubble substrate adjacent to a coral reef bommie with an average temperature of 27.2 °C. Aquarium-conditioned tetrapods were maintained at simulated mid-shelf reef 10-year average temperatures of 23.7–26.2 °C. Aquaria seawater was pumped from the ocean in Cleveland Bay, Australia (19.2181°S, 146.9222°E), pre-filtered, stored, further 4 µm filtered, and then fed into partially recirculating aquaria. Immediately prior to the annual 2018 mass-spawning event, tetrapods were collected from the reef, returned to AIMS, and maintained in aquaria for 10 days until larval settlement competency was established through standard assays [26]. Spawning and larval rearing information are located in the Supplementary Information (SI). A settlement choice experiment was conducted using 24 replicate tanks (50 L, receiving flow-through filtered seawater), with each tank containing three tetrapods from either reef- or aquarium-conditioned treatment, one from each conditioning time point (originally deployed 1, 2 or 3 months prior to settlement). This resulted in 12 tanks per conditioning type (Fig. 1). A fourth unconditioned tetrapod was added to each tank as a control after a two-day rinse in filtered seawater. 15-day-old A. tenuis coral larvae (*n* ~ 350) were transferred into each tank and left for 24 h to allow settlement before the tetrapods were removed for imaging (Nikon D810) to count the number of settled larvae (Randall et al. unpublished). Biofilms were subsequently removed from the tetrapods by scraping the surface with a sterile scalpel blade. Sampling was done separately on the surfaces, crevices, and bottoms of each tetrapod. The biomass was placed in cryotubes and immediately preserved in liquid nitrogen before storage at −75 °C until DNA extraction. Seawater was sampled from lines feeding the tanks (*n* = 3) and selected aquaria containing reef-conditioned (*n* = 3) and aquarium-conditioned (*n* = 3) tetrapods, with 5 L per sample filtered onto individual 0.2 µm Sterivex filters (Millipore/Merck) and stored at −75 °C. To enable differentiation of the biofilm community from the microbiome of settled A. tenuis, five replicate samples of 20 larvae were frozen in liquid nitrogen and stored at −75 °C pending DNA extraction.

### DNA extraction, sequencing and bioinformatics

Water sample DNA extractions were performed using a standard sterivex extraction protocol [27], while biofilm and coral larvae samples were extracted using the DNeasy® Ultraclean® Microbial kit (Qiagen). 16S and 18S rRNA amplicon sequencing was completed at the Ramaciotti Centre for Genomics on the Miseq platform (Illumina). Detailed methods are provided in the SI.

### Statistical analyses

*Acropora tenuis* larval settlement success was quantified for each tetrapod by dividing the total number of larvae settled on a tetrapod by the total sum of all settled larvae within that tank (Table S1). Tetrapods were subsequently grouped into low, medium and high-settlement categories based on histogram distributions (Figs. S1 and S2). This resulted in the following low, medium and high settlement categories for aquarium-conditioned tetrapods: 0–32%, 33–62%, and 63–100% (Fig. S1A) and for reef-conditioned tetrapods: 0–32%, 33–55%, and 56–100% (Fig. S2A). Read counts were pooled from the surface, crevices, and bottom to obtain one sample for each tetrapod. Non-metric Multi-Dimensional Scaling (nMDS) based on Bray-Curtis dissimilarity was conducted on the log transformed Amplicon Sequence Variants (ASVs) read counts using the R package vegan [28] to visualise partitioning of prokaryotic and eukaryotic communities according to sample type and time. PERMANOVA was calculated with vegan’s adonis function for the 16S and 18S rRNA data [28, 29]. Based on the results of the initial PERMANOVA, Pairwise PERMANOVA was performed for all datasets (16S and 18S rRNA aquarium and reef) independently comparing settlement category (low, medium, high) and conditioning time (1, 2 and 3 months) using pairwiseAdonis [29]. For the 16S and 18S rRNA reef datasets, the medium and high-settlement biofilms did not differ significantly (*p* > 0.05) and were therefore combined for a settlement range of 32.14–78.70%, representing high-settlement (Fig. S2B). Heatmaps were generated to visualise relative abundances across prokaryotic samples at the family taxonomic level in the reef and aquarium biofilms using pheatmap [30] and bar plots were generated to visualise relative abundances across eukaryotic samples at NBCI general taxonomic category level in Excel. Venn diagrams were created with the VennDiagram R package [31]. All analyses were performed in RStudio [32] unless otherwise indicated. Additional Chi-square statistics are provided in SI.

### Core microbiome

A core microbiome was defined for both aquarium and reef-conditioned 16S and 18S datasets across all time periods to remove transient prokaryotes and eukaryotes unlikely to be core members of the established biofilm. The core microbiome, filtered with OTUTable [33], included ASVs present in at least 2/3 of the sample sets and with a minimum 0.01% relative abundance [34]. The core microbiome was determined separately for each settlement level, allowing identification of settlement-specific ASVs. Once a core microbiome was obtained for each settlement category, settlement-specific core microbiome datasets were recombined for network analysis. The core microbiome’s relative abundance was further log-transformed using built in R log() functions [32] and the partitioning of prokaryotic and eukaryotic communities was visualised by settlement category and time with nMDS Bray-Curtis dissimilarity plots at the ASV level using vegan [28] (Figs. S1, S2).

### Network and community analysis

Co-occurrence networks were constructed separately for the core microbiome of 16S and 18S rRNA gene datasets and independently for both aquarium and reef datasets, using the network construction algorithm FlashWeave [35]. In these networks, microbial taxa are represented by nodes and their pairwise co-occurrences across samples are represented by the edges linking them. To unveil the network’s modular structure and identify groups of co-occurring microbes, we performed a modularity analysis on the weighted networks using the Netcarto algorithm (R package rnetcarto) [36].

Network nodes (ASVs) were represented as pie charts showing the distribution of settlement categories as relative fractions. Each ASV pie chart was created by dividing the sum of the normalised read counts for that ASV across either high, medium, or low-settlement biofilm samples by that ASV’s total abundance. To better visualise the distribution of larval settlement across network modules, we created a simplified network that collapsed modules into single nodes. Edge weights between nodes (each representing a module) were set to the number of connections between ASVs in different modules of the original networks. Nodes were represented as pie charts of relative fractions of each module by dividing the sum of ASV normalised counts per settlement category within that particular module by the total ASV counts in the module [37]. Furthermore, bridging nodes, identified as those with edges connecting different modules, were extracted using Networktools [38].

For the prokaryotic networks, node betweenness centrality and degree were calculated to identify nodes that had a significant influence on network connectivity in general and across modules, thus influencing microbial community structure [39]. Nodes that have high betweenness and low degrees are critical to network information flow because despite not being very connected, they are a prevalent steppingstone for transitioning to different parts of the network. Full details of network analyses, including details on Netcarto and FlashWeave algorithms, can be found in SI.

## Results

### Prokaryotic community structure differs across coral larval settlement categories

**Fig. 2:**
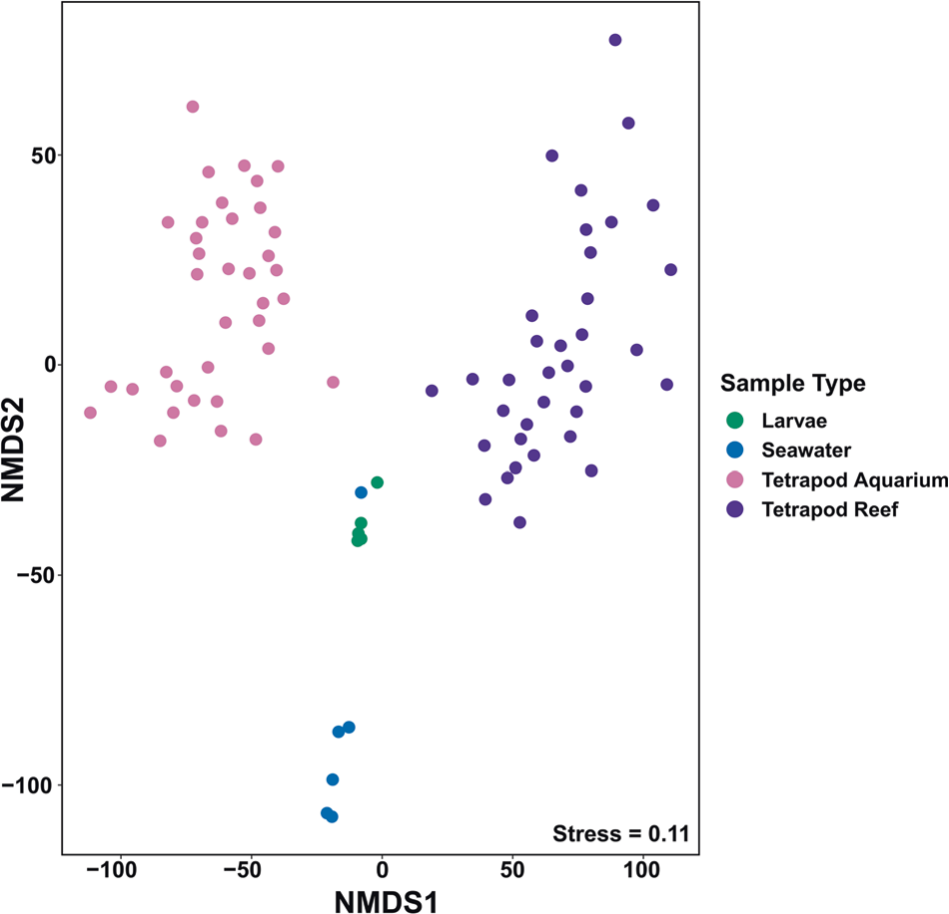
Prokaryotic communities are distinct across biofilms, seawater and larvae. nMDS highlighting the variation in 16S rRNA prokaryotic community composition across all sample types (Tetrapod Reef *N* = 35, Tetrapod Aquarium *N* = 35, Larvae *N* = 5, Seawater *N* = 6). Tetrapods from all time periods (1, 2 and 3 months) were grouped together.

**Fig. 3:**
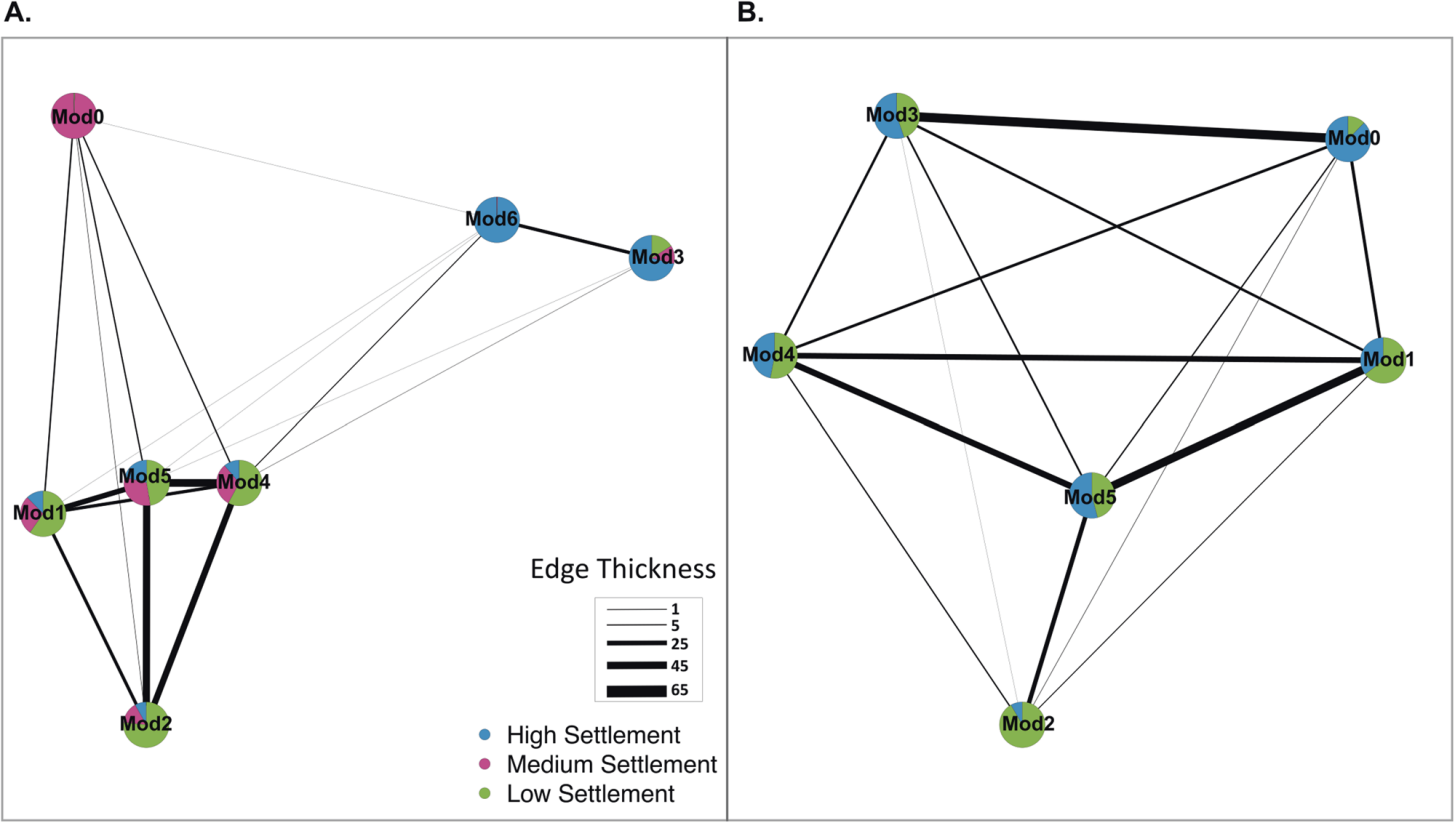
Modularity of prokaryotic co-occurrence networks in biofilms reflects the settlement success of coral larvae. Graphs show prokaryotic co-occurrence networks with overlaying settlement data. Simplified networks were created for prokaryotic communities within (**A**) aquarium-conditioned biofilms and (**B**) reef-conditioned biofilms at all sampling time points. Each mega node in these simplified networks represents a module of ASVs collapsed into one group (as shown in Fig. S4). The edges represent connections between bridging ASVs that co-occur across different modules. Edge thickness reflects the total number of connections between modules. The pie chart nodes show the proportion of ASVs that are associated with high (blue), medium (purple), and low-settlement categories (green).

Tetrapods harboured diverse prokaryotic communities dominated by *Proteobacteria* (Fig. S3). Prokaryotic communities differed significantly across samples types (larvae, seawater, aquarium biofilms and reef biofilms) (*F*_3_ = 24.94, *p* < 0.001, Fig. 2). Since settlement success covaried with conditioning time (aquarium biofilms *X*^*2*^(df = 4, *n* = 36) = 22.91, *p* < 0.001; reef biofilms *X*^*2*^(df = 2, *n* = 36) = 11.03, *p* < 0.004), we focused on settlement success as the primary variable and grouped samples across the three conditioning times. Co-occurrence networks of aquarium and reef-conditioned prokaryotic biofilms were highly modular and structured according to coral larval settlement category (aquarium network modularity *M* = 0.72; reef network modularity *M* = 0.68) (Figs. 3A, B; S4, S5). ASVs, represented by nodes in the network, grouped tightly with other ASVs that were dominant within similar settlement categories, leading to the development of predominantly single settlement-category modules (Fig. 3). For example, in the aquarium network, module 0 represents a predominantly medium-settlement community, with 99.3% of its ASVs only found in biofilms promoting medium-settlement (Fig. 3A). Similarly, module 2 in both the aquarium and reef networks represent low-settlement communities with 76.6% and 91.6% of their ASVs only found in low-settlement biofilms, respectively (Fig. 3A, B). Modules 6 and 0 contained 99.5% and 87.7% of ASVs associated with high settlement in the aquarium and reef networks, respectively (Fig. 2).

ASVs in modules 1, 3, 4 and 5 across both networks were found across multiple settlement-categories (Fig. 3A, B) and predominantly belonged to the families *Rhodobacteraceae*, *Flavobacteriaceae* and *Alteromondaceae* (Fig. S6). *Rhodobacteraceae* was the most abundant microbial family across all settlement-categories (Fig. S6).

**Fig. 4:**
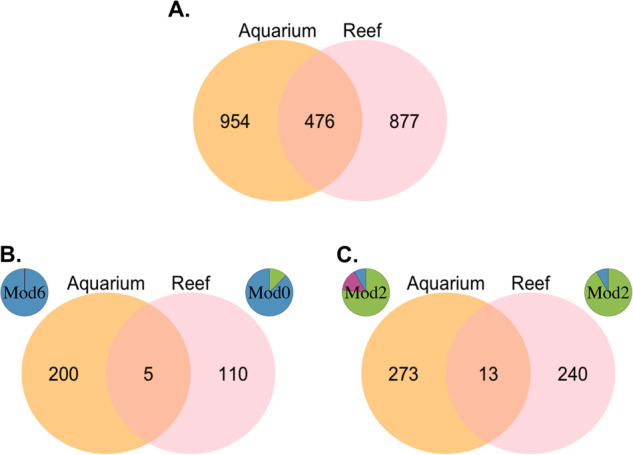
Prokaryotic ASV distribution across aquarium- and reef-conditioned biofilms at all sampling time points. **A** Total, **B** ASVs found in predominately high-settlement modules, **C** ASVs found in predominately low-settlement modules. Each network’s corresponding high- and low-settlement module is placed to its respective network (Aquarium-dataset is orange and reef-dataset is pink). The module pie charts depict the proportion of ASVs associated with high- (blue), medium- (purple), and low-settlement categories (green).

The aquarium biofilm core microbiome comprised 891 ASVs in high-settlement, 1,020 in medium-settlement, and 848 in low-settlement biofilms. Reef-conditioned core microbiome biofilms comprised 1,083 ASVs in high-settlement and 1,041 in low-settlement. In total, 476 ASVs were shared between both networks (Fig. 4A). Only five of these were shared between the aquarium and reef high-settlement modules: two *Rhodobacteraceae*, one *Micavibrionaceae*, one unassigned SAR324 (*Deltaproteobacteria*) and one unassigned KI89A (*Gammaproteobacteria*) (Fig. 4B), suggesting that potential settlement-inducing cues may originate from different taxa in aquarium and reef conditions. Furthermore, 13 ASVs were shared between low-settlement modules of both networks, and belonged to the taxonomic families *Cyclobacteriaceae*, *Xenococcaceae*, *Lentisphaeraceae*, *Kangiellaceae*, *Cellvibrionaceae*, *Rhodobacteraceae*, and *Sneathiellaceae*, as well as unassigned OM190 (*Planctomycetes*) and unassigned *Gammaproteobacteria* (Fig. 4C).

**Fig. 5:**
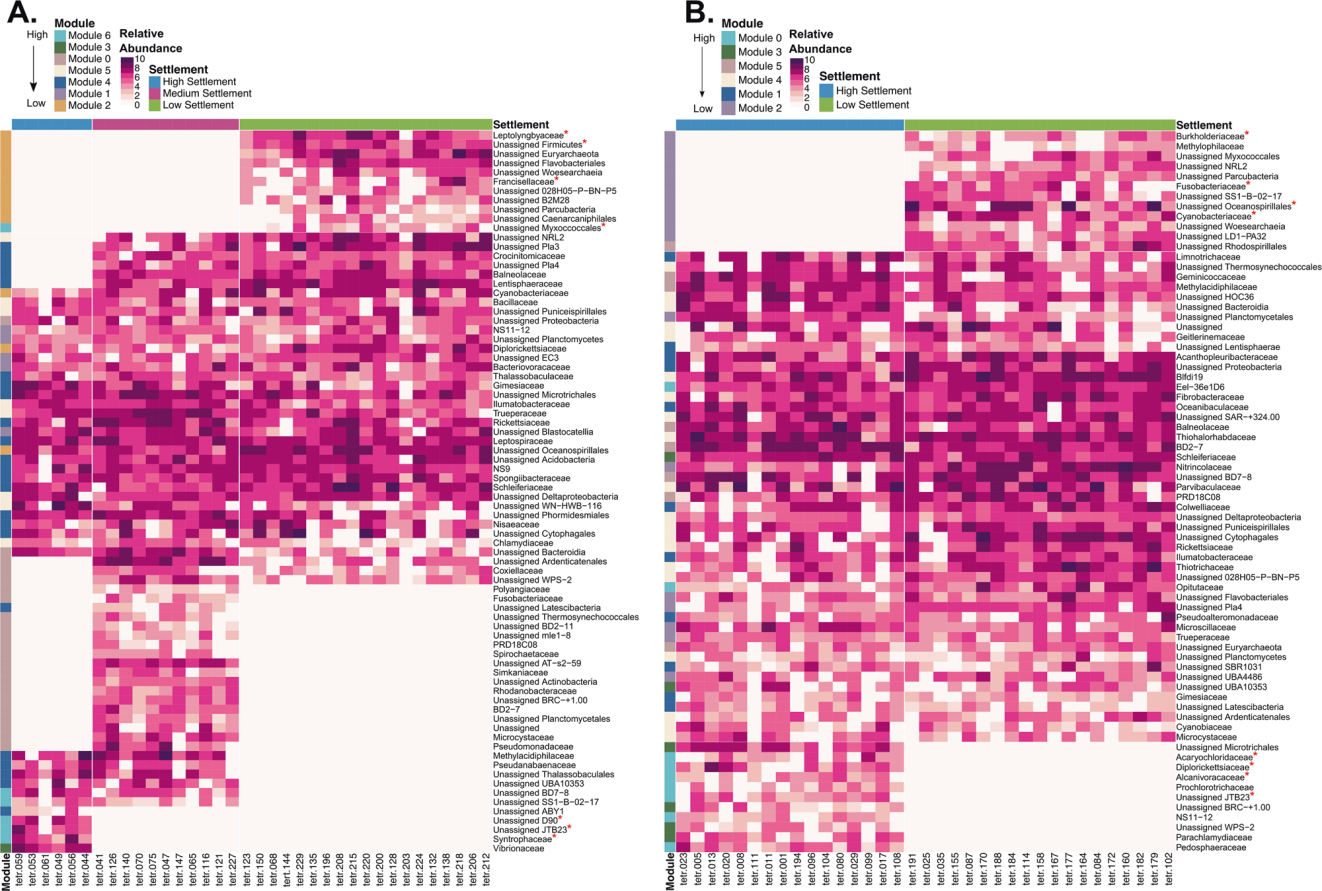
The distinct composition of aquarium and reef microbial biofilms at different larval settlement levels is reflected across rare prokaryotic families. Log transformed relative abundance of rare taxa (<0.1% relative abundance of 16 S rRNA gene sequencing reads) is shown at the family level (right) across biofilms samples within high (blue), medium (purple), and low-settlement (green) categories (top) for aquarium (**A**; *N* = 35) and reef (**B**; *N* = 35) datasets at all sampling time points. Corresponding modules for each family where it was present at the highest abundance are shown on the left. Families identified as containing potential inducers and inhibitors are labelled with a red asterisk. The module legend and biofilm samples are ordered based on their level of settlement (from highest to lowest).

A small subset of 146 microbial families were found exclusively in high-settlement modules, comprising mostly rare taxa (<0.1% relative abundance). This includes *Syntrophaceae*, unassigned D90 (*Gammaproteobacteria*) family, and unassigned *Myxococcales* family in the aquarium network (Fig. 5A); and *Acaryochloridaceae*, *Alcanivoracaceae*, *Diplorickettsiaceae* and NS11-12 (*Bacteroidetes*) in the reef network (Fig. 5B). The only microbial families shared between both networks and found exclusively in high or low-settlement samples belonged to JTB23 (*Gammaproteobacteria*) and *Woesearchaeia*, respectively. There were 11 families found exclusively in low-settlement samples in the aquarium network including *Francisellaceae*, *Leptolyngbyaceae*, and unassigned *Firmicutes* family (Fig. 5A) and 13 families in the reef network including, *Burkholderiaceae*, *Cyanobacteriaceae*, *Fusobacteriaceae*, *Paraspirulinaceae* and an unassigned *Oceanospirillales* family (Fig. 5B). ASVs belonging to these taxonomic families were further characterised using BLAST and improved taxonomic resolution was only found for 5 of the above ASVs (Table S2).

### Specific prokaryotes play an important role in linking settlement-inducing biofilm modules within the network

In general, both networks had common bridging node ASVs (i.e., nodes linking different modules), with *Rhodobacteraceae* being the most dominant family in both networks and mostly connecting mixed-settlement modules (Table S3). There was no overlap at the ASV level connecting high-settlement to mixed-settlement modules and only one ASV connected from mixed-settlement to low-settlement (Table S3). A diverse range of bridging node families connected low-settlement modules to the remaining nodes in both networks (Table S4). Additionally, an unassigned *Gammaproteobacteria* ASV connected a mixed-settlement module to a low-settlement module in the aquarium network, but the same ASV connected a high-settlement module in the reef network.

**Fig. 6:**
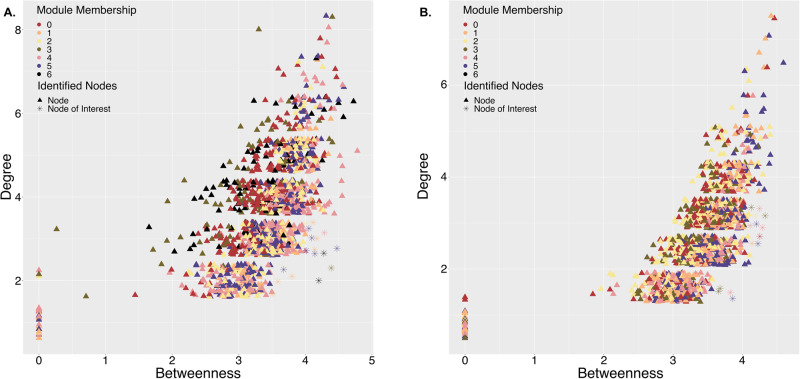
Prokaryotic network metrics showing node betweenness and degree. Comparison of node betweenness versus degree in the 16S rRNA gene network from (**A**) aquarium- and (**B**) reef-conditioned biofilms across all timepoints. Each triangle represents an individual node. The nodes with the highest betweenness values and lowest degrees are labelled as nodes of interest.

Numerous ASVs within mixed-settlement modules formed bridges to high-settlement modules in both networks (Table S4) and nodes of interest were narrowed down using betweenness and degree network metrics. Bridging nodes with high-betweenness (present in 3–5 pathways) and low-degree (connected to 2–4 other nodes) were of particular interest as they may represent prevalent stepping stones for network pathways between low-medium-high settlement-microbial communities. These nodes could thus represent ASVs having the potential to alter the composition of biofilm microbial assemblages resembling low-settlement communities in ways that might bring them closer to high-settlement ones. In the aquarium network, nodes of interest belonged primarily to *Rhodobacteraceae*, with the remaining nodes representing a wide range of taxa, including a *Thiohalorhabdaceae* ASV in high-settlement module 6 (Fig. 6A; Table S5A). This node represented the only ASV within module 6 that was found in medium-settlement biofilms instead of just high-settlement, and connected module 6 with mixed and medium-settlement modules (Fig. 3A). Furthermore, within the reef network, 14 bridging nodes with high-betweenness and low-degree were found facilitating connections between mixed and settlement-specific modules (Table S5B). Bridging connections in the reef network predominately occurred between high-settlement modules 3 and 5 and low-settlement modules 2 and 4 (Fig. 3B). These connections consisted of diverse microbial families between high-settlement modules and between low-settlement modules (Fig. 6B, Table S5B).

### A variety of eukaryotic taxa dominate high-settlement communities and do not consist of a single taxonomic group

**Fig. 7:**
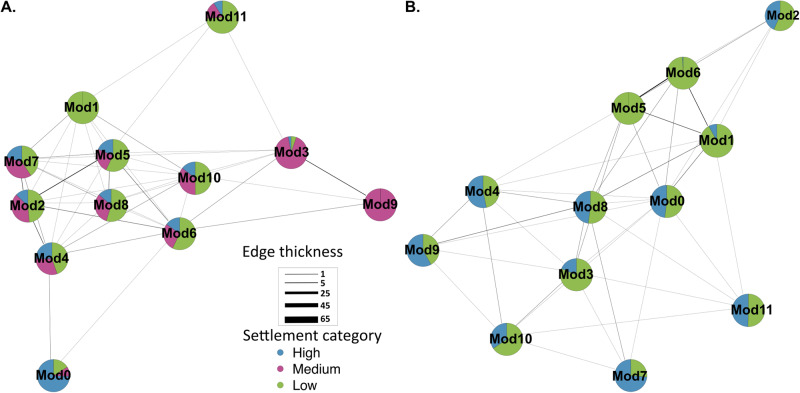
Modularity of eukaryotic co-occurrence networks in biofilms reflects the settlement success of coral larvae. Graphs show eukaryotic co-occurrence networks with overlaying settlement data. Simplified networks were created for eukaryotic communities within (**A**) aquarium-conditioned biofilms and (**B**) reef-conditioned biofilms across all timepoints. Each mega node in these simplified networks represents a module of ASVs collapsed into one group (as shown in Fig S4). The edges represent connections between bridging ASVs that co-occur across different modules. Edge thickness reflects the total number of connections between modules. Nodes are represented by pie charts showing the proportion of ASVs that are associated with high- (blue), medium- (purple), and low-settlement categories (green).

**Fig. 8:**
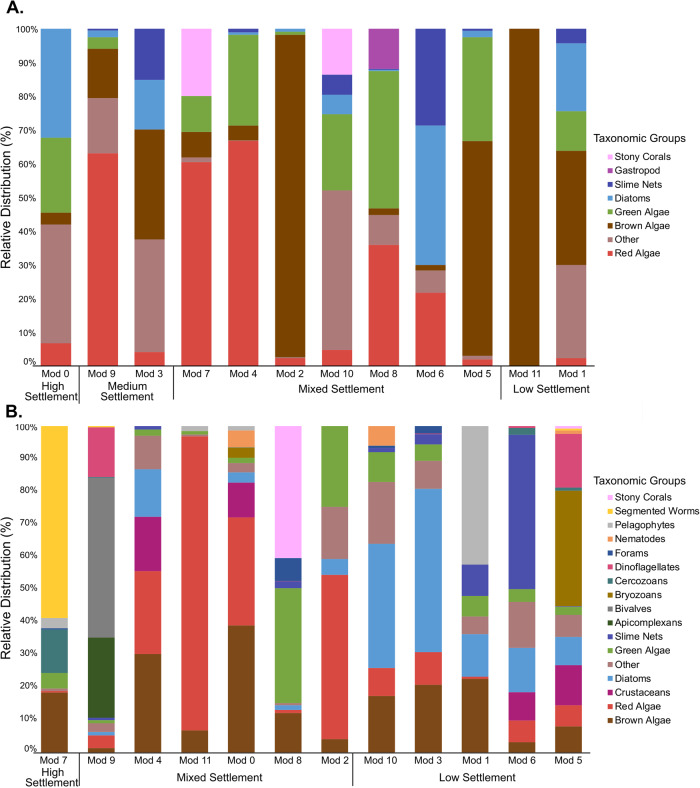
Distribution of eukaryotic groups within network modules. (**A**) aquarium- and (**B**) reef-conditioned biofilms across all sampling timepoints. Taxa representing <1% of the communities are grouped as “Other”. In the category Red Algae, all taxa were classified as CCA.

To assess the potential involvement of eukaryotes in coral larval recruitment, we analysed the 18S rRNA sequence data extracted from the biofilms in the larval choice experiments. Due to the short read lengths (average 177 bp) and low taxonomic resolution this provides, analyses of eukaryotic taxa were limited to broader categories than the prokaryotic data. However, abundance data needs to be interpreted with caution because there may be variation in 18S copy numbers that may introduce bias to abundance counts [40]. While this bias can be normalised using a corrective factor, the exact copy numbers are not known for the diversity of taxa identified here. Therefore, we decided not to correct the 18S copy number so that we would not introduce additional bias. As in the prokaryotic communities, the eukaryotic communities differed significantly across sample types (larvae, seawater, aquarium tetrapods and reef tetrapods) (*F*_3_ = 16.97, *p* < 0.001 Fig. S7). Eukaryotic co-occurrence networks were also highly structured by settlement category (aquarium network modularity *M* = 0.71; reef network modularity *M* = 0.72) (Fig. 7A, B; Fig. S4). As found in the prokaryotic biofilm communities, specific settlement-level modules comprised a variety of eukaryotic taxa rather than being dominated by any one taxonomic group (Fig. 8). Despite CCA showing highest relative abundance in high-settlement biofilm samples (Fig. S8), it was not found exclusively within high-settlement modules for either network (Fig. 8). Rather, CCA-derived ASVs were relatively more abundant in aquarium and reef mixed-settlement modules (Fig. 8; Table S6). Furthermore, brown algae and diatoms were most abundant in mixed-settlement modules in both aquarium and reef networks, as well as the low-settlement reef network modules (Fig. 8; Table S6). A small percentage of brown algae and diatom ASVs were exclusive to low-settlement biofilms, and while the majority were found across all settlement-categories, they were most abundant in low-settlement biofilms (Fig. S7; Table S6).

## Discussion

### Candidate prokaryotic inducers and inhibitors found in settlement-exclusive modules

Prokaryotic and eukaryotic co-occurrence networks derived from both reef and aquarium-conditioned biofilms were highly modular, and individual modules were associated with different coral larval settlement-inducing categories. High-settlement modules from both prokaryotic networks (reef and aquarium) were not dominated by any particular highly abundant microbial taxa, rather they were comprised of very diverse communities including many rare taxa. Furthermore, high-settlement modules contained taxa that are known to induce settlement in other marine invertebrate larvae. For example, *Myxococcales*, a bacterial order previously identified within biofilms that promotes scallop *Argopecten purpuratus* larval settlement [41], was found exclusively in high-settlement modules of the aquarium network. Similarly, an unassigned *D90* (*Gammaproteobacteria*) ASV, highly similar to *Granulosicoccus sp*., was observed exclusively in high-settlement aquarium biofilms. *Granulosicoccus* has been previously associated with CCA species that induced sea urchin larval settlement [42].

Potential inducers found in the reef high-settlement module include an *Alcanivoracaceae* ASV, which was exclusive to high-settlement biofilms. This microbial family was previously identified as an indicator species for the *Pocillopora acuta* coral recruit microbiome [43]. This *Alcanivoracaceae* ASV was 100% identical to the genus *Acaryochloris*, known to encode a bmp1 gene homologue thought to be involved in TBP biosynthesis [44]. TBP is a non-quorum signalling compound isolated from *Pseudoalteromonas* [45] that induces settlement and metamorphosis in larvae of some coral species [1, 17, 19, 25]. Although TBP is only found at very low concentrations naturally and does not consistently induce settlement and metamorphosis [46], our results add to mounting evidence supporting *Acaryochloris* as a taxon promoting settlement in *A. tenuis* larvae.

*Pseudoalteromonas sp*. induce settlement of a range of marine invertebrates (including corals) [47]. For example, *Pseudoaltermonas rubra* (strain #1783) isolated from CCA *Hydrolithon reinboldii*, was recently found to induce larvae of the brooding coral *Leptastrea purpurea* in mono- and mixed-species biofilms [25]. Reef and aquarium biofilms contained differing ASVs in an exclusively high-settlement family classified within *JTB23* (*Gammaproteobacteria*), where *Pseudoalteromonas* was the aquarium network’s ASV’s closest relative. Along with *Pseudoalteromonas*, Petersen et al. found that *Pseudovibrio denitrificans* (strain #1792) induced the highest levels of *L. purpurea* settlement in mono- and mixed-biofilms [25]. We identified two ASVs closely related to this strain in our reef high-settlement biofilms, which were located within the high-settlement portions of two mixed-settlement modules. These ASVs were also both bridging nodes connecting to other high-settlement nodes across the network, making them potential candidates for promoting settlement, directly or by facilitating the transition to a microbial community conducive of high settlement.

Aquarium and reef biofilms also shared ASVs exclusively found in low-settlement biofilms. For example, an unassigned *Cyclobacteriaceae* ASV found in both biofilms was highly similar to *Reichenbachiella agariperforans*, which has previously been reported as a potential inhibitor of larval settlement in the tube worm *Galeolaria hystrix* [48]. *Rhodobacteraceae, Sneathiella limimaris* and *Alcanivoracaceae* have also been reported in high abundance in biofilms that did not induce *G. hystrix* settlement [48]. In our study, a *Sneathiellaceae* ASV with high sequence similarity to *Sneathiella limimaris* was found in aquarium and reef-exclusively low-settlement modules. Additionally, an unassigned *Oceanospirillales* ASV, which is highly similar to *Alcanivorax sp*., was found only in reef low-settlement modules. Furthermore, a *Pleurocapsa* (*Xenococcaceae*) ASV was shared in both networks in low-settlement modules. This species was previously found to be highly abundant in biofilms that were conditioned under natural and acute anthropogenic stressors, including poor water quality, and was associated with no coral recruitment for some *Pocilloporidae* and *Acroporidae* corals [49].

Many prokaryotic taxa in biofilms associated with high-settlement were found at very low abundances, supporting previous hypotheses that low-abundance taxa may be capable of inducing larval settlement [21, 50]. Rare taxa (<1% relative abundance) often show positive interactions with each other in developing biofilms [51] via cross-feeding [52] and sharing metabolites [53], and can play an integral role in shaping biofilm communities [54]. The addition of these rare taxa in a multi-species community could create a community niche with new interrelated functions [55] that may support pathways and functions necessary to induce larval settlement. Such functions associated with high larval settlement could be related to the production of carbohydrates, amino acids and derivatives, or protein metabolism, all of which have been found in biofilms that induce *H. elegans* larval settlement [56]. The high diversity of rare microorganisms within high-settlement modules across the reef and aquarium networks suggests a level of functional redundancy, where the environment selects different microorganisms capable of performing similar functions. Finally, it is important to note that the analysis was performed on entire tetrapods, while settlement levels were not uniform on the entire surface of each tetrapod (Randall, personal communication). Further research should endeavour to identify the density of microbes needed for the candidates identified here as rare taxa to successfully attract larvae. While microbial community was the focus on the analysis presented here, it is also possible that the variation in conditioning time influenced other features of the substrate, influencing the microbiome and/or settlement. For example, longer conditioning time potentially could result in more physical or biological erosion of the substrate, creating more microhabitats on the surface and thus influence the microbial community composition and/or settlement. However, no visible physical differences were observed amongst tetrapods of differing conditioning times and given these short conditioning times, relative to erosion rates, we think this is unlikely to play a significant role in driving the observed patterns. In addition, microbial biofilms were only sampled after coral larval settlement, and therefore, cannot distinguish if the presence of settled larvae altered the microbial biofilm communities. Future studies can experimentally validate potential inducing taxa and additionally follow microbial biofilm composition through time including just prior to and after larval settlement to test if the presence of larvae affects the microbial biofilm community composition.

### Prokaryotic taxa that form the bridge between mixed-settlement and high-settlement niches

Larval settlement was correlated with aquarium and reef-conditioned biofilm development, where primary and secondary colonisers were more abundant in low and mixed-settlement modules. The role of primary colonisers has been shown to transform the biofilm environment by providing functions such as nitrogen fixation [57], metabolite production [58], and modifying pH and oxygen content [58]. In both networks, *Rhodobacteraceae* were abundant primary colonisers, which are thought to shape biofilm structure using quorum-sensing via acylated homoserine lactones to enable cell-to-cell communication [59, 60]. Following primary colonisation there may be sufficient nutrients available for secondary colonisers to settle [58, 61]. In our networks, we identified members of the phyla *Actinobacteria*, *Cyanobacteria*, *Planctomycetes*, *Gammaproteobacteria*, and *Alphaproteobacteria* as secondary colonisers due to their high relative abundances in biofilms conditioned for two or more months and in the mixed-settlement modules, consistent with biofilm studies testing settlement of *Balanus amphitrite* [62] and *H. elegans* [63]. Microbial communities in mixed-settlement modules could represent secondary colonisers that are transforming the biofilm environment to one that supports colonisation of settlement inducing microorganisms found in the high-settlement modules. Understanding microbial biofilm succession is important because it gives insight into what types of microorganisms are needed in the biofilm community to create an environment (i.e., create necessary by-products, affect pH levels etc.) where settlement-inducing microorganisms can successfully survive.

Bridging nodes connecting mixed-settlement to high-settlement modules were diverse in taxonomy across both prokaryotic networks. The pathways between bridging nodes may suggest community succession between biofilms inducing differing coral larval settlement levels. Out of the 16 ASVs identified in the aquarium network with high-betweenness and low-degree, only one, a *Thiohalorhabdaceae* ASV, bridged between high-settlement module and mixed-/medium-settlement modules. This specific ASV could be important for facilitating high-settlement community niches since it was found across all settlement levels and connecting high-settlement to mixed-settlement modules. *Thiohalorhabdales* are sulfur oxidisers [64] involved in nitrogen cycling [65], that could provide a detoxified micro-environment [66] as well as nutrients to attract microbes promote settlement. Furthermore, *Rhodobacteraceae*, a common taxon in marine biofilms [59, 67], was the most dominant family to bridge modules of varying settlement levels, possibly due to its ability to shape biofilm communities with cell-to-cell communication during succession [59, 60]. The *Rhodobacteraceae* genus *Phaeobacter*, only found in our aquarium network, was the only node associated with low-settlement in an overall high-settlement community, and, therefore, may be a key species in facilitating transitions between communities of different settlement levels.

### CCA, brown algae and diatoms dominate high and low-settlement-inducing eukaryotic communities

Aquarium and reef biofilms hosted a diverse eukaryotic community, where the relative abundance of CCA was highest in high-settlement inducing biofilms, consistent with previous research showing CCA as an effective inducer of coral settlement for various coral species [14–17, 68–70]. While CCA-derived compounds 11-deoxyfistularin-3 and luminaolide induce settlement of *L. purpurea* larvae [46, 71], several studies have also shown involvement of CCA-associated microbes in settlement. For example, glycoglycerolipids and betaine lipids associated with the microbial biofilm on the CCA *Titanoderma prototypum* induce *Acropora cytherea* settlement [11]. Interestingly, CCA was not the dominant eukaryotic taxa in high-settlement modules from either reef or aquarium biofilms; instead, high-settlement modules consisted of a broad range of taxonomic groups. CCA was also found in medium and low-settlement biofilms, which indicates that its presence alone had a limited impact on settlement in those biofilms. This could be attributed to potential community differences of the prokaryotes associated with CCA at differing settlement levels, differences in the CCA species themselves, or due to the co-occurrence of other settlement-inhibiting microorganisms within these biofilms.

Within low-settlement biofilms of the aquarium and reef eukaryotic communities, brown algae were the most abundant eukaryote and were found in mixed-settlement modules with predominately low-settlement percentages. Macroalgae can inhibit larval settlement of the barnacle *B. Amphitrite* [72] by producing exudates that can be easily consumed by microorganisms as a carbon source, potentially attracting opportunistic pathogenic microorganisms [73–76]. Brown algae also inhibited settlement of the corals *Porites astreoides* [77, 78] and *Acropora sp*. [79–81]. For example, the brown alga genus *Lobophora* can inhibit *A. hyacinthus* and *A. gemmifera* larval settlement through waterborne allelochemicals up to one metre spatially [80, 82]. However, other brown algae, such as *Lobophora variegata*, can induce settlement of *A. millepora* [79]. Similarly, *Ectocarpales siliculosus* was shown to inhibit larval settlement of the barnacle *Semibalanus balanoides*, but not the barnacle *A. amphitrite* [83]. Although *L. variegata* were not found in our dataset, *Ectocarpales* was the most abundant order of brown algae in low-settlement modules in both networks and was predominantly found in the low fraction of mixed-settlement modules. While the effect of brown algae, and more specifically *Ectocarpales*, on *A. tenuis* coral larval settlement is largely unknown, its presence across samples in all settlement levels suggests that its presence does not necessarily deter settlement.

Diatoms had the second-highest relative abundance in low-settlement biofilms in both networks and were predominantly found in mixed-settlement modules. Common diatoms found in biofilms include the genera *Navicula*, *Amphora*, *Nitzschia*, *Pleurosigma* and *Thalassionema* [84] and their effect on marine larval settlement responses appear to be species-specific [23]. For example, the diatoms *Coconeis* and *Navicula ramoissima* can induce settlement of the barnacle *A. amphitrite*, while genera *Achnanthes*, *Navicula*, and *Nitzschia* inhibit settlement. Diatoms also inhibited the settlement of the polychaete *H. elegans* [23, 85, 86], the bryozoan *Bugula* [87], and the barnacle *S. balanoides* [88]. The most prevalent diatom orders in both networks were *Naviculaires*, *Bacillariales*, and *Thalassiophysales* and their roles in coral larval settlement, specifically for *A. tenuis*, are largely unknown. These diatom orders were previously found in association with the organic matrix formed by brown algae [89], and here, we find these diatoms in mixed-settlement modules alongside brown algae in both settlement networks. Therefore, diatoms within these biofilms may be associated with the presence of brown algae, but whether or not they have an inhibitory effect on *A. tenuis* coral larvae needs further elaboration.

### Concluding remarks

Here, we used a co-occurrence network analysis to elucidate the complexities of microbial biofilms and showed that microbial biofilms correlating with different levels of larval settlement in *A. tenuis* have distinct and diverse prokaryotic and eukaryotic communities. The identification of specific taxa in high-settlement modules and putative inhibiting taxa in low-settlement modules, as well as *Rhodobacteraceae* as potential transitionary microorganisms, further narrow down microorganisms of interest for future settlement validation experiments. Taxa belonging to prokaryotic families of interest identified here are likely culturable based on the previous culturing success of individual strains within these families [59, 90–97]. Furthermore, overlaying functional information may help inform genome-guided cultivation for taxa that prove to be more challenging to culture [98]. In addition, our results show that the involvement of CCA, brown algae and diatoms on *A. tenuis* larval settlement warrants further investigation. Future research should aim to cultivate these prokaryotic and eukaryotic taxa to experimentally validate their roles in larval settlement, as has been successfully undertaken with other biofilm systems [54–56, 59, 68, 93–96]. Cultivability is also important for aquaculture and restoration because microbially-derived inducive cues need to be easily applied to settlement surfaces and produced at scale to meet coral aquaculture needs.

Considering the diverse array of microorganisms contributing to high-settlement modules, further research is needed to determine whether individual taxa or specific microbial functions, distributed across different microbial lineages are responsible for initiating coral larval settlement. Selecting microorganisms based on a shared metabolism rather than specific taxa, for reef restoration purposes, would provide significant flexibility for the design of microbial cues that can be used in coral aquaculture.

### Supplementary information

Supplementary Material

## Data Availability

The datasets analysed during the current study are available in the NCBI Sequence Read Archive (SRA) (https://www.ncbi.nlm.nih.gov/sra) under the BioProject accession number PRJNA978954.
