# Supplementary material for: Linking differences in microbial network structure with changes in coral larval settlement
Source: ISME Commun. 2023 Oct 21;3:114. doi: 10.1038/s43705-023-00320-x (PMC10590418; doi:10.1038/s43705-023-00320-x)
Supplement: Supplementary file 1 — Supplementary Material [file 43705_2023_320_MOESM1_ESM.docx]

**Supplementary Material for the paper:**

**Linking transitions in microbial community structure with changes in coral larval settlement**

Authors: Abigail C. Turnlund^1^, Inka Vanwonterghem^1^, Emmanuelle S. Botté^2,3^, Carly J. Randall^3^, Christine Giuliano^3^, Lisa Kam^3^, Sara Bell^3^, Paul O’Brien^1^, Andrew P. Negri^3^, Nicole S. Webster^1,3,4^, and Miguel Lurgi^5^

^1^The University of Queensland, School of Chemistry and Molecular Biosciences, Australian Centre for Ecogenomics, St Lucia, QLD, 4072, Australia

^2^Centre for Marine Science and Innovation, School of Biological, Earth and Environmental Sciences, University of New South Wales, Sydney, NSW, Australia

^3^Australian Institute of Marine Science, Townsville, QLD, Australia

^4^Australian Antarctic Division, Department of Climate Change, Energy, the Environment and Water, Kingston, Australia.

^5^Department of Biosciences, Swansea University, Swansea, SA2 8PP, UK

*Supplementary Methods*

*Coral spawning and larval rearing*

Seven adult *A. tenuis* coral colonies were collected from Backnumbers reef, GBR on November 24^th^ 2018 and spawned on November 28^th^ 2018, five days after the full moon at 19:25 (50 min after sunset). Spawning and larval rearing followed standard protocols, as detailed in Pollock et al. (1).

*DNA Extractions*

70 mg of biofilm material and 20 mg of *A. tenuis* larvae were used per sample and extractions followed the manufacturer’s instructions using the DNeasy® Ultraclean® Microbial kit (Qiagen), except for an additional five second vortex after sample material was added to the solution and then placement into the FastPrep 24 (MP Biomedicals) for 40 seconds at four m s^-1^ to lyse the cells by both mechanical and chemical action.

*DNA Quality Control*

The purity of DNA in the samples was assessed by measuring the 260/280 and 260/230 (absorbance in nm) ratios. The Qubit dsDNA HS assay kit was used on the Qubit 3.0 Fluorometer (Invitrogen) to accurately quantify DNA concentration, according to the manufacturer’s instructions. Samples were stored at -20˚C.

*Sequencing and bioinformatics*

The primers 515F – 806R (Forward primer: 5’-GTGCCAGCMGCCGCGGTA-3’; Reverse Primer: 5’-GGACTACHVGGGTWTCTAAT-3’) (2) were used to amplify the 16S rRNA gene, and 1391f-EukBr (Forward primer: 5’-GTACACACCGCCCGTC-3’ and reverse primer: 5’-TGATCCTTCTGCAGGTTCACCTAC-3’) (3, 4) was used to amplify the 18S rRNA gene. 18S and 16S rRNA gene amplicons were sequenced to produce 2 x 150 bp and 2 x 250 bp paired end reads respectively. Raw sequences were demultiplexed and the resulting fastq files were processed with Qiime2 (version 2018.11) using DADA2 to denoise (5). For the 16S rRNA gene amplicons, forward sequences were truncated to 240 bp and the reverse sequences truncated to 230 bp. Sequencing depth was consistent across samples within each sample type, i.e., seawater, larvae and biofilms (Table S6). For the 18S rRNA gene amplicons, the first 10 bp from the forward and reverse reads were removed to eliminate reduced quality bases. Both 16S rRNA and 18S rRNA sequences were classified using the Silva database (version 132, 99_majority taxonomy), and contaminants were removed through filtering of the core microbiome. In addition, mitochondria, eukaryote, and chloroplast sequences were removed from the final 16S rRNA ASV tables. The taxonomy of both 16S and 18S rRNA ASVs of interest were further clarified with BLASTn, using the top matches (minimum 80% identity and a minimum 95% sequence alignment) for the ASVs at the genus level.

*Chi-square Tests*

Chi-square contingency table tests were performed to analyse if conditioning time and settlement-category covaried with the chisq.test() function in the R Stats package (6). Contingency tables were constructed separately for aquarium- and reef-conditioned tetrapods for separate Chi-square values. For both datasets, contingency tables were constructed with tetrapod counts per conditioning time and settlement-category.

*FlashWeave*

FlashWeave implements a local neighbourhood search optimisation algorithm on the normalized species (ASVs in our case) counts to link nodes (representing microbial species) with direct associations in a co-occurrence network (7). FlashWeave first normalizes the microbial data with centred log-ratio transformation (8) (Eq. 1). In Equation 1, *g*(*s_i_)* is the geometric mean of all abundances in samples (*s_i_*), *p* is the total number of ASVs, and *x_ij_* is the abundance of ASV *j* in sample *s_i_* (7).

$\mathrm{clr}\left( x_{ij} \right)=log\frac{x_{ij}}{g\left( s_{i} \right)} , with g(s_{i})=\left[ \prod_{I=1}^{p} x_{il} \right]^{\frac{1}{p}}$ (1)

The data thus normalised is used to infer links with a local optimal neighbourhood search for modules by orderings ASVs (i.e., nodes in the network) by degree (defined as the number of associations they have) with the learn_network function. The node with the largest degree is the first node added to a tentative group with all of its directly associated neighbours (7). All of the remaining nodes in the network are continually added to the group, one by one, and tested against other nodes already in the group with partial correlation tests. If the test is not significant (p>0.05), the node is rejected from the group (7). Once all nodes are tested, the first nodes added to the group (the node with the highest degree and all of its direct associations) are checked against the rest of the final group and eliminated if not significant (7). Nodes are then linked if they are found in each other’s individual neighbourhood or module, and a weight measure is assigned to each node which is equal to the node’s degree (i.e., the number of links it has) (7). We used the learn_network function’s sensitivity mode parameter, and this parameter dictates which method is used to test significancy, with the default using discretised mutual information tests and the sensitive mode using partial correlation tests, between the normalised read count data of a given pair of ASVs across samples (7). Different heuristic methods such as Max-k, false discovery rate adjustment, feedforward, and fast-elimination are applied in order to increase efficiency of the local to global learning framework (7). This algorithm assumes casual sufficiency, so there are no hidden variables and only using the variables provided can influence links within the network (9). Full details of the FlashWeave algorithm can be found in Tackmann *et al.* (7).

*Modularity analysis*

The Netcarto modularity algorithm relies on simulated annealing optimization to obtain partitions of nodes that maximise modularity *M* (Eq. 2) (10). This partitioning procedure results in clusters of nodes (i.e., modules) that have more connections to other nodes within the cluster than to nodes outside the cluster (11-14),

$M = \sum_{S=1}^{N_{m}} \left[ \frac{l_{S}}{L}-\left( \frac{d_{S}}{2L} \right)^{2} \right]$ (2)

where *N_m_* is the number of modules, *L* is the total number of edges in the network, *l_s_* is the number of edges between nodes in module *s*, and *d_s_* refers to the sum of the degree (i.e. number of links) of all nodes in module *s* (10, 15).

*Network Metrics*

Node degree refers to the number of edges connected to the node. Betweenness centrality (*x_i_*) is a measure that quantifies the centrality of a node in the network by counting the number of times a node is traversed by all paths between every pair of nodes in the network, i.e. the higher the betweenness, the more essential a node is in relaying information across a network (12) (Eq. 3):

$x_{i}=\sum_{st} \frac{n_{st}^{i}}{g_{st}}$ (3)

where *n^i^_st_* is the number of shortest paths between nodes *s* and *t* that pass through node *I*, and *g_st_* is the total number of shortest paths between nodes *s* and *t* (12)*.* Node betweenness and degree were quantified using the igraph R package (16) in both reef and aquarium tetrapod networks. Node betweenness values were log transformed to reduce the skewness of the data (17).

*Module settlement relative fractions*

The distribution of families across modules was determined by taking the sum of all the normalised ASVs belonging to that family within that module and dividing the abundance of a family within a specific module by the total abundance of that family in all modules, while the relative fraction was further standardised using Hellinger’s transformation before beta diversity.

*Supplementary Tables and Figures:*

***Table S1: Percent coral larval settlement per tetrapod.***

| Tetrapod | Treatment | Conditioning Location | Settlement (%) |
| --- | --- | --- | --- |
| 1 | 3-mo deployment | Field | 45.5 |
| 5 | 3-mo deployment | Field | 66.5 |
| 8 | 3-mo deployment | Field | 51 |
| 11 | 3-mo deployment | Field | 48 |
| 13 | 3-mo deployment | Field | 56 |
| 17 | 3-mo deployment | Field | 34.5 |
| 20 | 3-mo deployment | Field | 55.5 |
| 23 | 3-mo deployment | Field | 78.5 |
| 25 | 3-mo deployment | Field | 29 |
| 29 | 3-mo deployment | Field | 38 |
| 35 | 3-mo deployment | Field | 28 |
| 80 | 2-mo deployment | Field | 39 |
| 84 | 2-mo deployment | Field | 16 |
| 87 | 2-mo deployment | Field | 27.5 |
| 96 | 2-mo deployment | Field | 44.5 |
| 99 | 2-mo deployment | Field | 34.5 |
| 102 | 2-mo deployment | Field | 9 |
| 104 | 2-mo deployment | Field | 44 |
| 108 | 2-mo deployment | Field | 34.5 |
| 111 | 2-mo deployment | Field | 48.5 |
| 114 | 2-mo deployment | Field | 26 |
| 155 | 2-mo deployment | Field | 28 |
| 158 | 2-mo deployment | Field | 24 |
| 160 | 1-mo deployment | Field | 15 |
| 164 | 1-mo deployment | Field | 17 |
| 167 | 1-mo deployment | Field | 20.5 |
| 170 | 1-mo deployment | Field | 27 |
| 172 | 1-mo deployment | Field | 15 |
| 177 | 1-mo deployment | Field | 20.5 |
| 179 | 1-mo deployment | Field | 9 |
| 182 | 1-mo deployment | Field | 12 |
| 184 | 1-mo deployment | Field | 26.5 |
| 188 | 1-mo deployment | Field | 27 |
| 191 | 1-mo deployment | Field | 29 |
| 194 | 1-mo deployment | Field | 45.5 |
| 41 | 3-mo deployment | Lab | 51.5 |
| 44 | 3-mo deployment | Lab | 63.5 |
| 47 | 3-mo deployment | Lab | 44 |
| 49 | 3-mo deployment | Lab | 70.5 |
| 53 | 3-mo deployment | Lab | 88.5 |
| 56 | 3-mo deployment | Lab | 66 |
| 59 | 3-mo deployment | Lab | 92 |
| 61 | 3-mo deployment | Lab | 79.5 |
| 65 | 3-mo deployment | Lab | 38.5 |
| 68 | 3-mo deployment | Lab | 24 |
| 70 | 3-mo deployment | Lab | 48.5 |
| 75 | 3-mo deployment | Lab | 47 |
| 116 | 2-mo deployment | Lab | 38.5 |
| 121 | 2-mo deployment | Lab | 38 |
| 123 | 2-mo deployment | Lab | 28.5 |
| 126 | 2-mo deployment | Lab | 51.5 |
| 128 | 2-mo deployment | Lab | 8 |
| 132 | 2-mo deployment | Lab | 4.5 |
| 135 | 2-mo deployment | Lab | 21 |
| 138 | 2-mo deployment | Lab | 4 |
| 140 | 2-mo deployment | Lab | 50 |
| 144 | 2-mo deployment | Lab | 24 |
| 147 | 2-mo deployment | Lab | 39 |
| 150 | 2-mo deployment | Lab | 28 |
| 196 | 1-mo deployment | Lab | 14 |
| 200 | 1-mo deployment | Lab | 10 |
| 203 | 1-mo deployment | Lab | 7 |
| 206 | 1-mo deployment | Lab | 3.5 |
| 208 | 1-mo deployment | Lab | 11.5 |
| 212 | 1-mo deployment | Lab | 3.5 |
| 215 | 1-mo deployment | Lab | 11 |
| 218 | 1-mo deployment | Lab | 4 |
| 220 | 1-mo deployment | Lab | 10.5 |
| 224 | 1-mo deployment | Lab | 5 |
| 227 | 1-mo deployment | Lab | 36.5 |
| 229 | 1-mo deployment | Lab | 22.5 |

***Table S2: NCBI classification of selected ASVs of interest, showing top matches with genus-level classification*** (minimum 80% identity and a minimum 95% sequence alignment).

| ASV Taxonomic Family | Accession  No. | Bit  Score | E  Value | %  Similarity | NCBI Taxonomy |
| --- | --- | --- | --- | --- | --- |
| *Unassigned D90* | MT464839.1 | 407 | 4E-109 | 95.67 | *Granulosicoccus sp.* |
| *Alcanivoracaceae* | LC190498.1 | 468 | 2E-127 | 100 | *Acaryochloris sp.* |
| *Unassigned Cyclobacteriaceae* | NR113854.1 | 385 | 2E-102 | 94.07 | *Reichenbachiella agariperforans* |
| *Sneathiellaceae* | LT797158.1 | 451 | 2E-122 | 98.81 | *Sneathiella limimaris* |
| *Unassigned Oceanospirillales* | MT453890.1 | 390 | 4E-104 | 94.47 | *Alcanivorax sp.* |

***Table S3: Limited ASVs overlap between high- and low-settlement prokaryotic aquarium and reef network modules***

| Module Location | Shared ASVs | Associated Families | Most Abundant Taxa |
| --- | --- | --- | --- |
| *Both Networks* | 83 | 41 | *Rhodobacteraceae* |
| High-Settlement Modules | 0 | - | - |
| Low-Settlement Modules | 1 | 1 | Unassigned OM190 (*Planctomycetes*) |

***Table S4: Bridging nodes connecting to high and low-settlement from mixed-modules in prokaryotic aquarium and reef networks.***

| Network | Total bridging edges | | High-Settlement Modules | | | Low-Settlement Modules |
| --- | --- | --- | --- | --- | --- | --- |
|  | |  | Total edges | Associated Families | Taxonomy | Families Shared across both networks |
| Aquarium | 280 | | 28 | 3 | *Rhodobacteraceae*  *Flavobacteriaceae*  *Microtriachaceae* | 98 |
| Reef | 330 | | 127 | 58 | *i.e., Opitutaceae*  *Stappiaceae* |  |

**Table S5: Node degree and betweenness.** Taxonomic family, degree, and betweenness from bridging nodes (ASVs) of interest identified from the (A) 16S rRNA prokaryotic aquarium-network and (B) 16S rRNA prokaryotic reef-network. Nodes of interest were defined as having high-betweenness (3-5) and low-degree (2-4). Node AD is a node of interest for inducing Acropora tenuis coral larval settlement.

**A.**

| Node Label | Taxonomic Family | Degree | Betweenness |
| --- | --- | --- | --- |
| AA | Unassigned Eurycoccales (Oxyphotobacteria) | 3 | 4.47 |
| AB | Rhodobacteraceae | 2 | 4.39 |
| AC | Rhodobacteraceae | 3 | 4.28 |
| AD | Thiohalorhabdaceae | 3 | 4.28 |
| AE | Unassigned SS1-B-02-17 (Lentisphaerae) | 2 | 4.2 |
| AF | Unassigned NB1-j (Deltaproteobacteria) | 3 | 4.17 |
| AG | Microtrichaceae | 3 | 4.14 |
| AH | Rhodobacteraceae | 3 | 4.14 |
| AI | Rhizobiaceae | 3 | 4.12 |
| AJ | Flavobacteriaceae | 3 | 4.1 |
| AK | Unassigned Gammaproteobacteria | 3 | 4.05 |
| AL | Rhodobacteraceae | 3 | 4.01 |
| AM | Unassigned OM190 (Planctomycetes) | 3 | 4 |
| AN | Acanthopleuribacteraceae | 3 | 4 |
| AO | Parvularculaceae | 2 | 3.8 |
| AP | Rhodobacteraceae | 2 | 3.68 |
| AQ | Kordiimonadaceae | 2 | 3.59 |
| AR | Calditrichaceae | 2 | 3.51 |

**B.**

| Node Label | Taxonomic Family | Degree | Betweenness |
| --- | --- | --- | --- |
| RA | Unassigned Bacteria | 4 | 4.33 |
| RB | Rhodothermaceae | 4 | 4.29 |
| RC | Opitutaceae | 3 | 4.25 |
| RD | Unassigned Bacteroidia | 4 | 4.25 |
| RE | Leptospiraceae | 3 | 4.23 |
| RF | Stappiaceae | 4 | 4.2 |
| RG | Pseudohongiellaceae | 4 | 4.18 |
| RH | Unassigned Alphaproteobacteria | 3 | 4.14 |
| RI | Unassigned Gammaproteobacteria | 4 | 4.13 |
| RJ | Unassigned OM190 (Planctomycetes) | 4 | 4.11 |
| RK | Unassigned Eurycoccales (Oxyphotobacteria) | 2 | 3.86 |
| RL | Saprospiraceae | 2 | 3.83 |
| RM | Cryomorphaceae | 2 | 3.68 |
| RN | Oleiphilaceae | 2 | 3.66 |

***Table S6: Distribution of eukaryotic ASVs and corresponding relative abundances in settlement modules in the aquarium and reef networks.***

| Eukaryote | Sample Settlement-Category | Network | Module | Number of ASVs | Total Relative Abundance (%) |
| --- | --- | --- | --- | --- | --- |
| CCA | Mixed-Settlement | Aquarium | 4 | 7 | 31.4 |
|  |  |  | 7 | 8 | 40 |
|  |  | Reef | 0 | 2 | 53.9 |
|  |  |  | 11 | 4 | 21.7 |
|  | High-Settlement | Aquarium | 0 | 6 | 0.15 |
|  |  | Reef | 7 | 1 | 0.23 |
|  |  |  | 4 | 1 | 0.24 |
|  |  |  | 9 | 1 | 0.24 |
| Brown Algae | Mixed-Settlement | Aquarium | 2 | 10 | 48.5 |
|  |  |  | 5 | 11 | 28.8 |
|  |  | Reef | 0 | 6 | 28.68 |
|  |  |  | 3 | 5 | 19.82 |
|  | Low-Settlement | Aquarium | 1 | 5 | 1.2 |
|  |  |  | 11 | 1 | 0.03 |
|  |  | Reef | 0 | 1 | 0.49 |
|  |  |  | 4 | 3 | 0.83 |
|  |  |  | 5 | 4 | 1.24 |
| Diatoms | Mixed-Settlement | Aquarium | 6 | 9 | 75.9 |
|  |  | Reef | 3 | 4 | 60 |
|  | Low-Settlement | Aquarium | 1 | 4 | 5.4 |
|  |  | Reef | 1 | 7 | 2 |
|  |  |  | 2 | 1 | 0.23 |
|  |  |  | 5 | 5 | 1.7 |
|  |  |  | 6 | 6 | 1.6 |

***Table S7: 16S and 18S rRNA sequencing depth for different sample types***

| Sample Type | 16S rRNA minimum reads | 16S rRNA maximum reads | 18S rRNA minimum reads | 18S rRNA maximum reads |
| --- | --- | --- | --- | --- |
| Tetrapods | 24786 | 82539 | 3402 | 82699 |
| Larvae | 44769 | 74687 | 43879 | 67756 |
| Seawater | 74151 | 103084 | 19246 | 41878 |
| Negative Controls | 359 | 2453 | 16 | 113 |


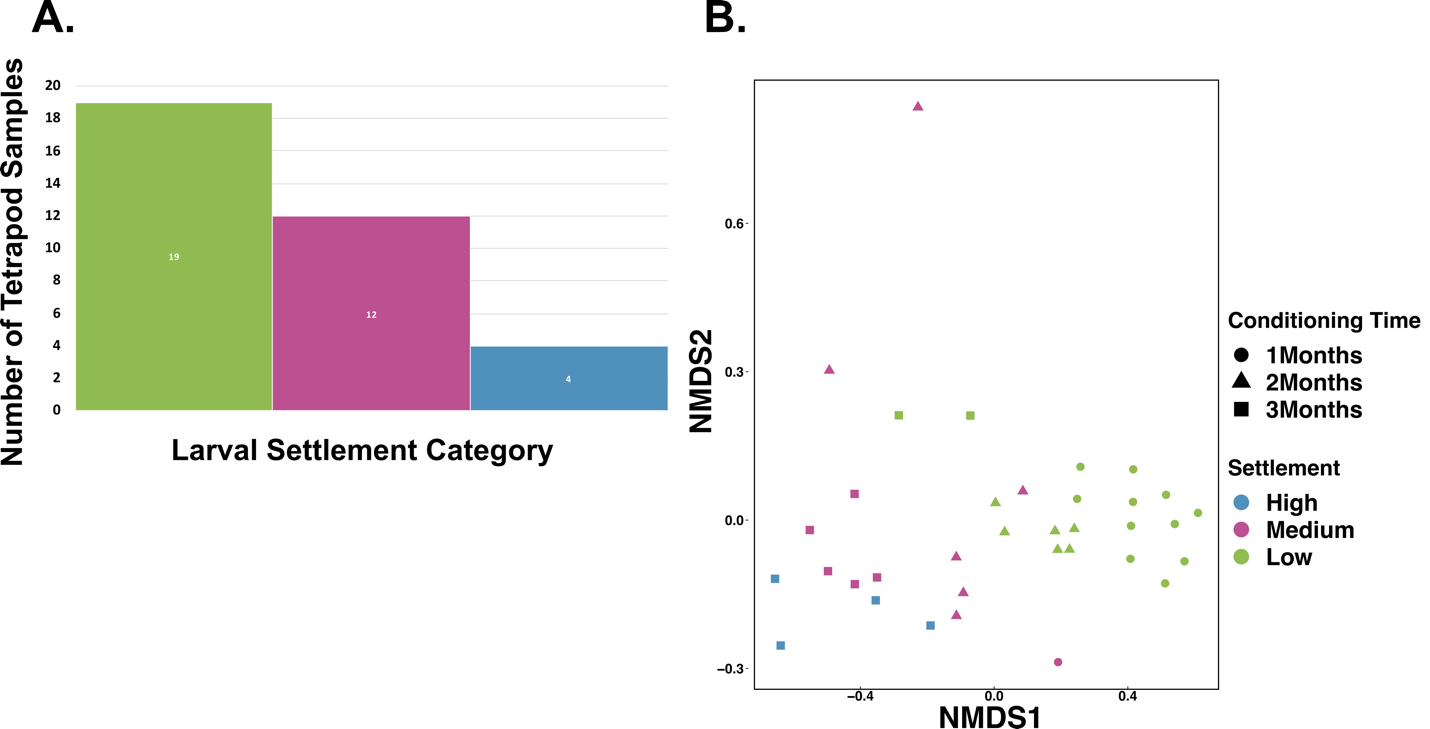


***Figure S1:***  ***16S rRNA*** ***microbial community composition across larval settlement categories for aquarium-conditioned tetrapods*.** (A) The distribution of *Acropora tenuis* larval settlement on aquarium-conditioned biofilms. The three categories were labeled to represent tetrapod biofilm samples associated with low (0 – 32%), medium (33 – 62%), and high (63-100%) settlement (N = 35). (B) NMDS plot of aquarium-conditioned biofilms, comparing the variation in microbial community composition across samples assigned to high (blue), medium (purple), and low (green) settlement categories and different conditioning time (1, 2, and 3 months).


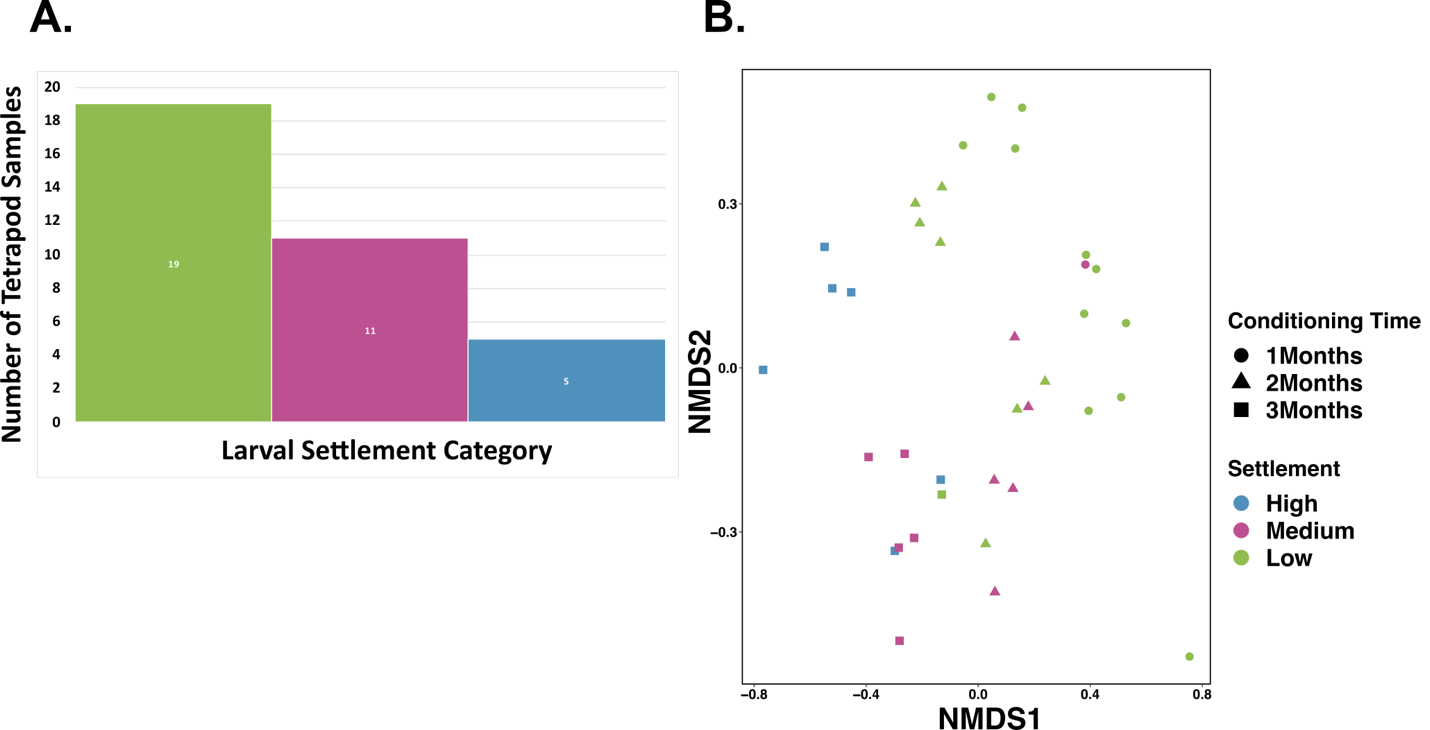


**Figure S2: 16S microbial community composition across larval settlement categories for reef-conditioned tetrapods**. (A) The distribution of Acropora tenuis larval settlement on reef-conditioned biofilms. The three categories were further labeled to represent tetrapod biofilm samples associated with low (0 – 32%), medium (33 – 55%), and high (56 – 100%) settlement. After PERMANOVA analysis of settlement level, the medium and high categories were combined to represent a single category called high-settlement (N = 35). (B) NMDS plot of reef-conditioned biofilms, comparing the variation in microbial community composition across samples assigned to high (blue), medium (purple), and low (green) settlement categories and across different conditioning times (1, 2, and 3 months).


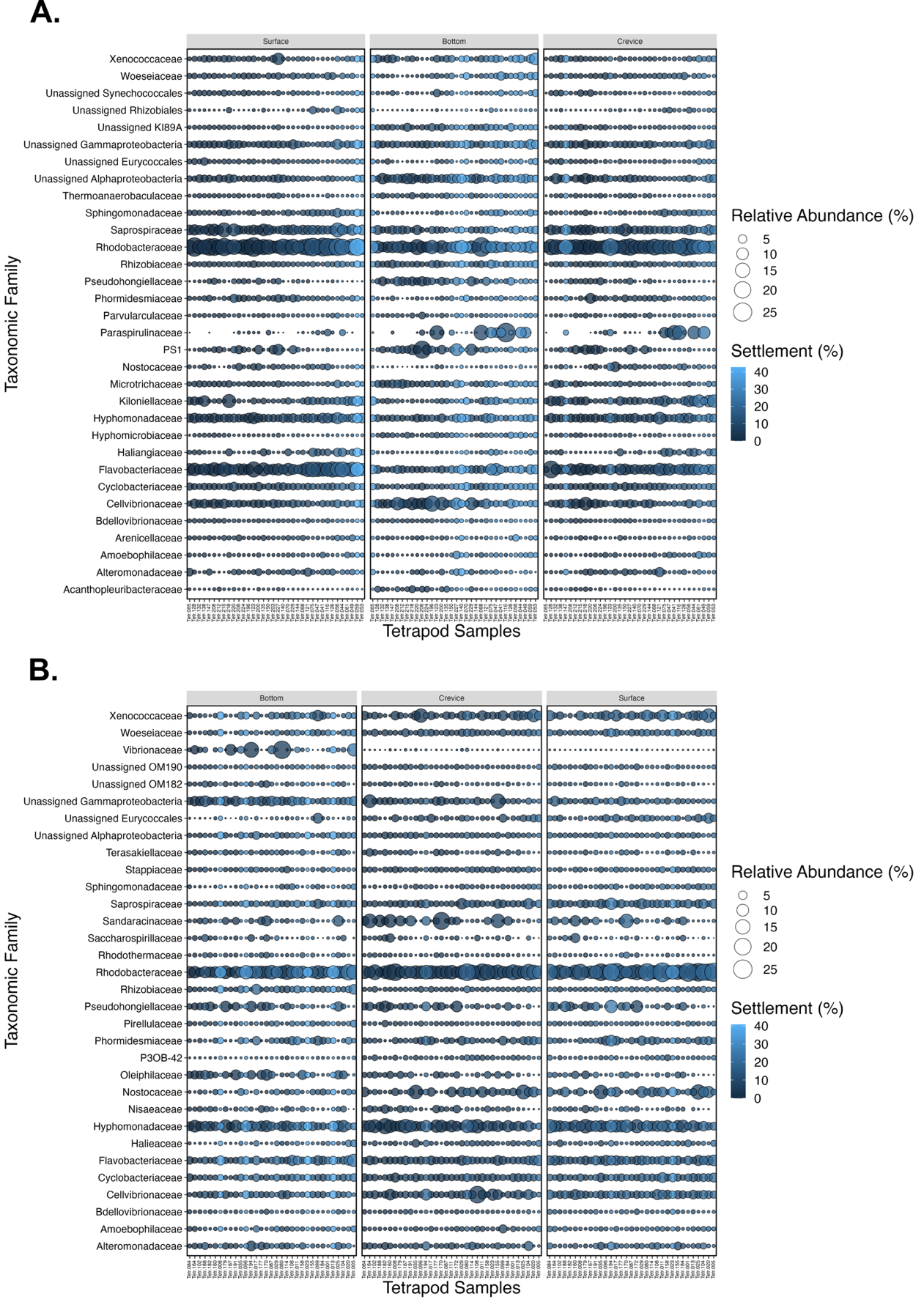


***Figure S3: Microbial community relative abundance across aquarium and reef-conditioned biofilms at different ecotypes.*** Prokaryotic taxonomic family relative abundance across (A) aquarium (N = 35) and (B) reef-conditioned biofilm samples (N = 35) at different ecotypes. Only taxonomic families that had a relative abundance greater than 1% across conditioning month categories were included. The size of the plot points correlates to the relative abundance and the color represents settlement percentage of *Acropora tenuis* larval settlement.


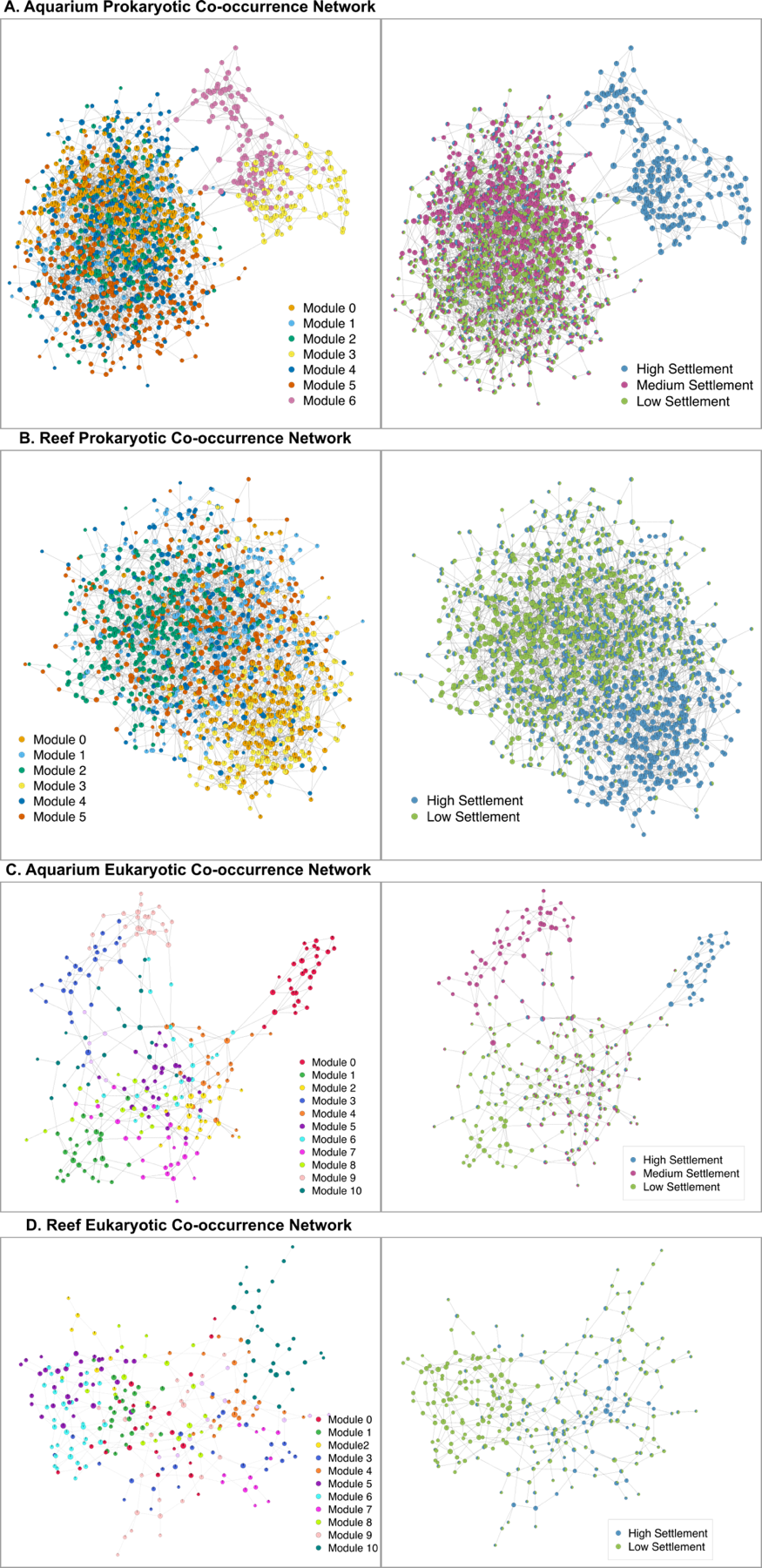


***Figure S4: Microbial co-occurrence networks.*** Co-occurrence network from prokaryotic aquarium (A), prokaryotic reef (B), eukaryotic aquarium (C), and eukaryotic reef-conditioned biofilms (D). For the networks on the left, each node represents a unique ASV, each edge represents a co-occurrence relationship between two ASVs, and node color represents ASV module membership through modules 0-6. Nodes of similar colors are tightly grouped together indicating the network is highly modular. For the networks on the right, it is the same network with node color representing distribution of ASVs by settlement category using pie charts, i.e high (blue), medium (purple), and low (green) settlement of *Acropora tenuis* larvae.


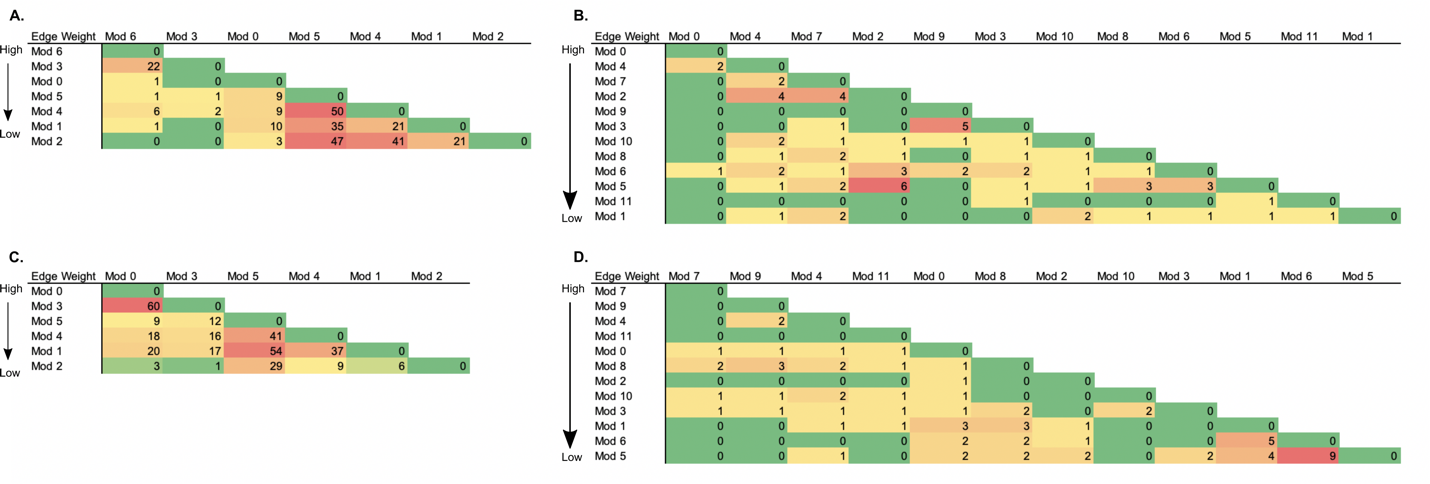


***Figure S5: Settlement Network weighted values.*** The number of connecting edges between modules in the (A) prokaryotic aquarium-conditioned biofilm network, (B) eukaryotic aquarium-conditioned biofilm network, (C) prokaryotic reef-conditioned biofilm network, and (D) eukaryotic reef-conditioned biofilm network.


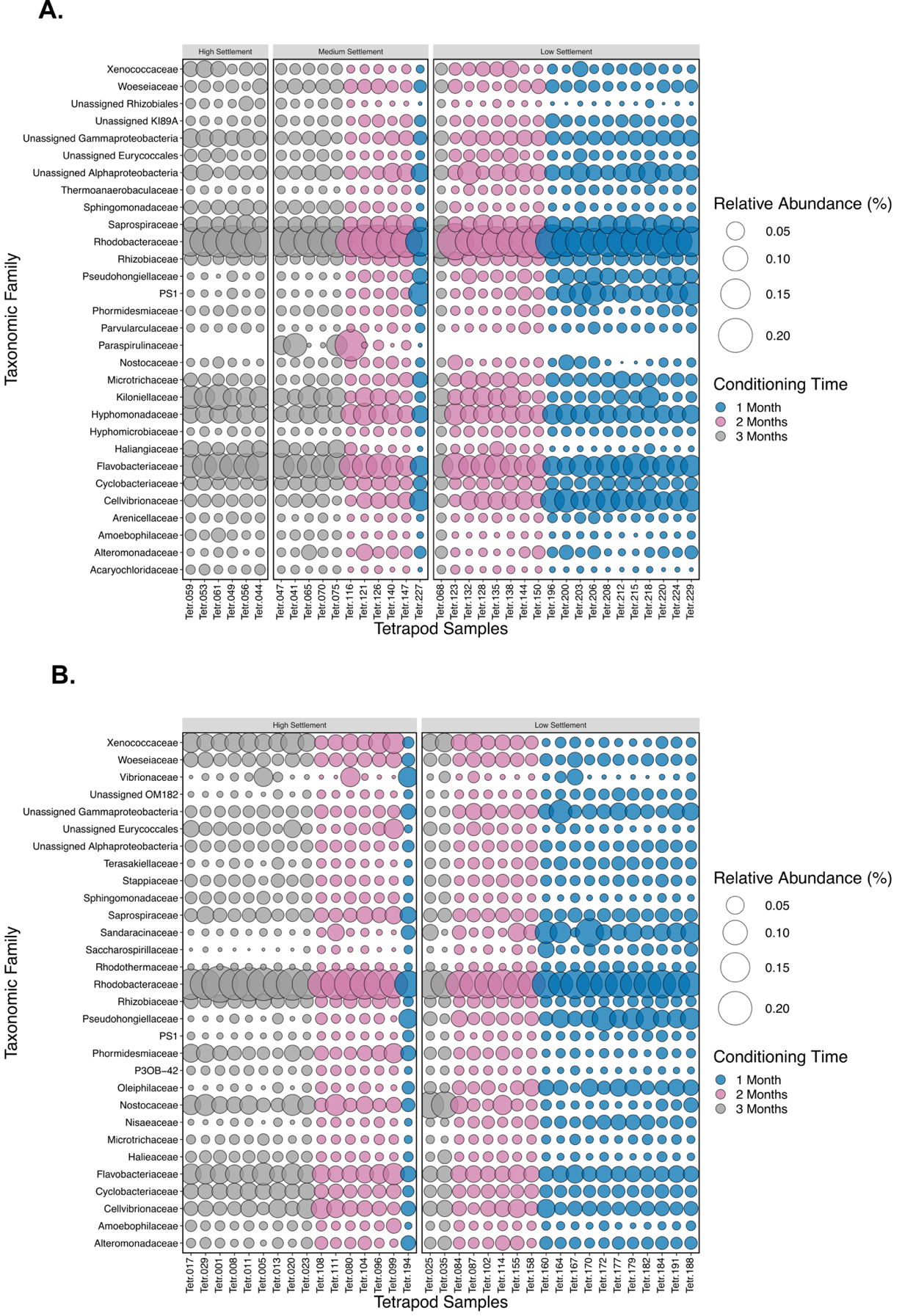


***Figure S6: Microbial community relative abundance across aquarium and reef-conditioned biofilms.*** Prokaryotic taxonomic family relative abundance across (A) aquarium (N = 35) and (B) reef-conditioned biofilm samples (N = 35) that different levels of *Acropora tenuis* larval settlement. Only taxonomic families that had a relative abundance greater than 1% across settlement categories were included. The size of the plot points correlates to the relative abundance and the color represents length of time conditioned (one, two, or three months).

***Figure S7. Eukaryotic communities are distinct between biofilms, seawater and larvae.*** nMDS highlighting the variation in 18S rRNA eukaryotic communities (p<0.001) across all sample types (Tetrapod Reef N = 35, Tetrapod Aquarium N = 35, Larvae N = 5, Seawater N =6). Tetrapods are separated based on location conditions, aquarium (pink) and reef (purple), and includes tetrapods from all time periods (1, 2, and 3 months).


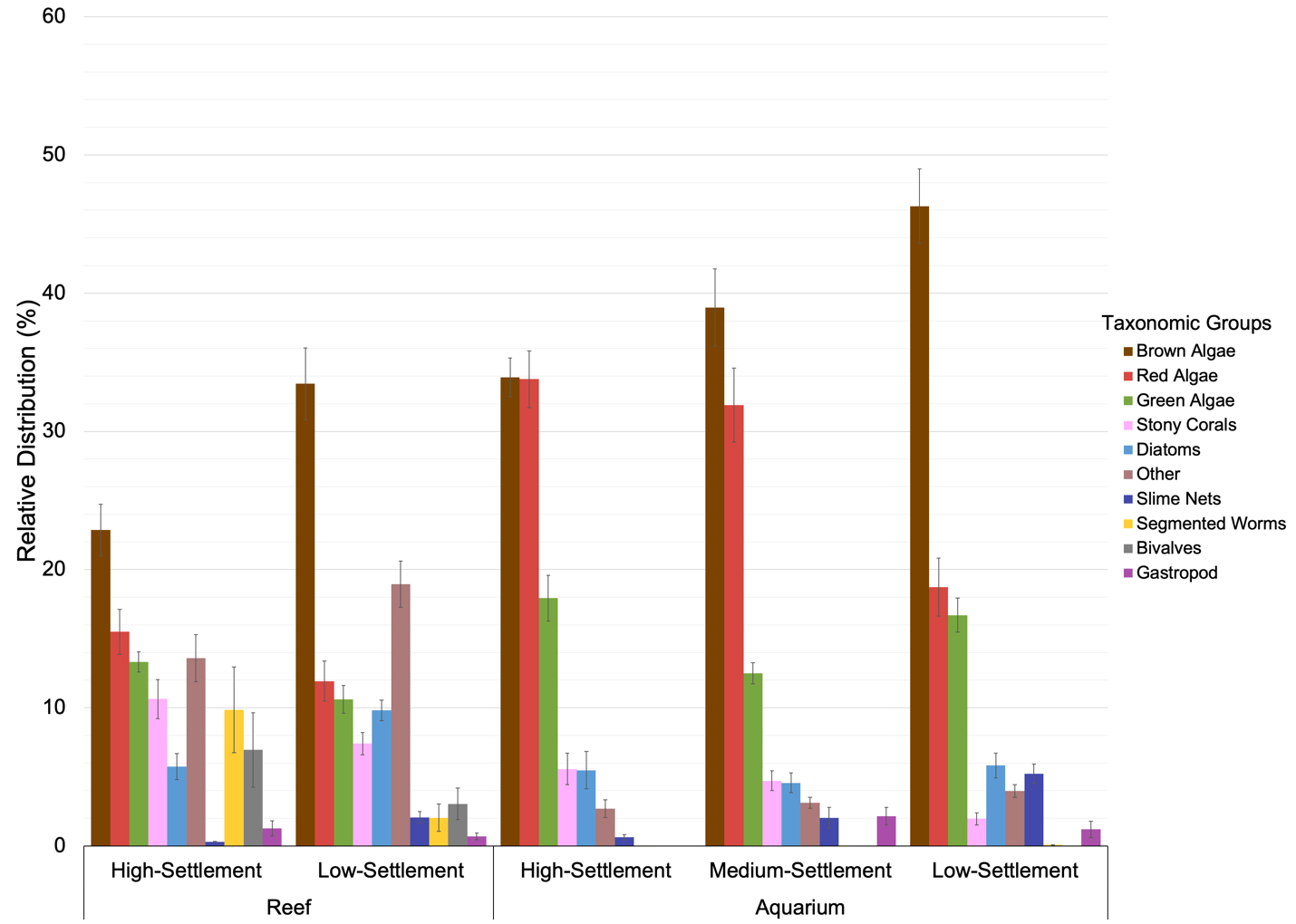


***Figure S8.*** ***Distribution of average relative abundance of eukaryotic groups within settlement-categories of reef-conditioned and aquarium-conditioned biofilm samples across all sampling timepoints.*** Taxa representing <1% of the communities are grouped as “Other”. In the category Red Algae, all taxa were classified as CCA. Error bars represent standard error. (Reef High-Settlement N = 16, Reef Low-Settlement N = 19, Aquarium High-Settlement N = 4, Aquarium Medium-Settlement N = 12, Aquarium Low-Settlement N = 19).

**References**

1. Pollock FJ, Katz SM, van de Water JA, Davies SW, Hein M, Torda G, et al. Coral larvae for restoration and research: a large-scale method for rearing Acropora millepora larvae, inducing settlement, and establishing symbiosis. PeerJ. 2017;5:e3732.

2. Caporaso JG, Lauber CL, Walters WA, Berg-Lyons D, Lozupone CA, Turnbaugh PJ, et al. Global patterns of 16S rRNA diversity at a depth of millions of sequences per sample. Proc Natl Acad Sci USA. 2011;108:4516-4522.

3. Amaral-Zettler LA, McCliment EA, Ducklow HW, Huse SM. A method for studying protistan diversity using massively parallel sequencing of V9 hypervariable regions of small-subunit ribosomal RNA genes. PLoS One. 2009;4(7):e6372.

4. Stoeck T, Bass D, Nebel M, Christen R, Jones MDM, Breiner HW, et al. Multiple marker parallel tag environmental DNA sequencing reveals a highly complex eukaryotic community in marine anoxic water. Mol Ecol. 2010;19(1):21-31.

5. Callahan BJ, McMurdie PJ, Rosen MJ, Han AW, Johnson AJA, Holmes SP. DADA2: High-resolution sample inference from Illumina amplicon data. Nat Methods. 2016;13(7):581-583.

6. Team RC. R: A Language and Environment for Statistical Computing. 2013.

7. Tackmann J, Matias Rodrigues JF, Von Mering C. Rapid inference of direct interactions in large-scale ecological networks from heterogeneous microbial sequencing data. Cell Syst. 2019;9(3):286-296.

8. Aitchison J. A new approach to null correlations of proportions. J Int Assoc Math Geol. 1981;13(2):175-189.

9. Aliferis CF, Statnikov A, Tsamardinos I, Mani S, Koutsoukos XD. Local causal and Markov blanket induction for causal discovery and feature selection for classification part II: analysis and extensions. J Mach Learn Res. 2010;11(1).

10. Girvan M, Newman MEJ. Community structure in social and biological networks. Proc Natl Acad Sci USA. 2002;99(12):7821-7826.

11. Faust K, Raes J. Microbial interactions: from networks to models. Nat Rev Microbiol. 2012;10(8):538-550.

12. Newman ME. Finding community structure in networks using the eigenvectors of matrices. Physical review E. 2006;74(3):036104.

13. Layeghifard M, Hwang DM, Guttman DS. Disentangling interactions in the microbiome: a network perspective. Trends in Microbiology. 2017;25(3):217-228.

14. Rezende EL, Albert EM, Fortuna MA, Bascompte J. Compartments in a marine food web associated with phylogeny, body mass, and habitat structure. Ecology Letters. 2009;12(8):779-788.

15. Bloomfield NJ, Knerr N, Encinas-Viso F. A comparison of network and clustering methods to detect biogeographical regions. Ecography. 2018;41(1):1-10.

16. Nepusz GCaT. The igraph software package for complex network research. InterJournal. 2006;Complex Systems:1965.

17. Matchado MS, Lauber M, Reitmeier S, Kacprowski T, Baumbach J, Haller D, et al. Network analysis methods for studying microbial communities: A mini review. Comput Struct Biotechnol J. 2021;19:2687-2698.
